# RNF157 attenuates CD4^+^ T cell-mediated autoimmune response by promoting HDAC1 ubiquitination and degradation

**DOI:** 10.7150/thno.86307

**Published:** 2023-06-19

**Authors:** Peng Wang, Jingjing Zhao, Yunke Tan, Junli Sheng, Shitong He, Yitian Chen, Dingnai Nie, Xiaolong You, Jinmei Luo, Yanling Zhang, Shengfeng Hu

**Affiliations:** 1 Department of Emergency Medicine, Sun Yat-sen Memorial Hospital, Sun Yat-sen University, Guangzhou, China.; 2 Department of Biotherapy, Sun Yat-sen University Cancer Center, Guangzhou, China.; 3 State Key Laboratory of Oncology in South China, Collaborative Innovation Center for Cancer Medicine, Sun Yat-sen University Cancer Center, Guangzhou, China.; 4 The Second Affiliated Hospital, The State Key Laboratory of Respiratory Disease, Guangdong Provincial Key Laboratory of Allergy & Clinical Immunology, Guangzhou Medical University, Guangzhou, China.; 5 Department of Internal Medicine, Medical Intensive Care Unit and Division of Respiratory Diseases, the Third Affiliated Hospital of Sun Yat-sen University, Guangzhou, China.; 6 Experimental Center of Teaching and Scientific Research, School of Laboratory Medicine and Biotechnology, Southern Medical University, Guangzhou, China.; 7 Department of Rheumatology and Clinical Immunology, Zhujiang Hospital, Southern Medical University, Guangzhou, China.

**Keywords:** CD4^+^ T cells, RNF157, HDAC1, Multiple sclerosis, Ubiquitination

## Abstract

**Background:** CD4^+^ T cells play an important role in body development and homeostasis. Quantitative and functional changes in CD4^+^ T cells result in abnormal immune responses, which lead to inflammation, cancer, or autoimmune diseases, such as multiple sclerosis (MS). Ubiquitination plays an essential role in the differentiation and functioning of CD4^+^ T cells. However, the function of several E3 ubiquitin ligases in CD4^+^ T cell differentiation and T cell-mediated pathological diseases remains unclear.

**Methods:** RNA sequencing data were analyzed to identify the E3 ubiquitin ligases that participate in the pathogenesis of MS. Furthermore, conditional knockout mice were generated. Specifically, flow cytometry, qPCR, western blot, CO-IP and cell transfer adoptive experiments were performed.

**Results:** In this study, we identified The RING finger 157 (RNF157) as a vital regulator of CD4^+^ T cell differentiation; it promoted Th1 differentiation but attenuated Th17 differentiation and CCR4 and CXCR3 expressions in CD4^+^ T cells, thereby limiting experimental autoimmune encephalomyelitis development. Mechanistically, RNF157 in CD4^+^ T cells targeted HDAC1 for K48-linked ubiquitination and degradation. Notably, RNF157 expression was significantly decreased and showed a significant negative correlation with RORγt expression in patients with MS.

**Conclusions:** Our study highlights the critical role of RNF157 in regulating CD4^+^ T cell functions in autoimmune diseases and suggests RNF157 as a potential target in adaptive immune responses against MS and other autoimmune disorders.

## Introduction

Multiple sclerosis (MS) is a chronic inflammatory, demyelinating, and neurodegenerative disease of the central nervous system (CNS). It is an autoimmune disease initiated by CD4 T helper (Th) cells specific for antigens in the myelin sheath 1-3. Experimental autoimmune encephalomyelitis (EAE) is a widely used animal model for MS 4. CD4^+^ T cells are the main cells involved in the acquired immune response and play an important role in body development and homeostasis. Upon T cell antigen receptor stimulation and the synergistic activity of cytokines, naive CD4^+^ T cells differentiate into distinct subsets, including Th1, Th2, Th17, and regulatory T (Treg) cells 5, 6. Among these subsets, Th1 cells produce IFN-γ as their signature cytokine, whereas Th17 cells produce their signature cytokine, IL-17^7^. Studies have shown that Th1 and Th17 cells may be involved in developing MS 8. In particular, Th17 cells are the main contributors to autoimmunity and tissue damage 9. By contrast, Treg cells represent immunosuppressive properties essential for regulating immune responses and maintaining peripheral tolerance 10. In addition, chemokines are a crucial component of the immune system. Activated Th cells express an array of inflammatory chemokine receptors (such as CCR4, CCR6, and CXCR3) synergistically recruiting immune cells to inflamed tissues 11. The dysregulation of chemokines has been linked to several pathological diseases, including MS 12.

Changes in different lineage-specific gene expressions cause the function and differentiation of Th cells. Specific transcriptional factors and epigenetic mechanisms are crucial in lineage-specific gene expression 13. Histone deacetylase 1 (HDAC1) is a key epigenetic regulator that plays a vital role in the function and differentiation of Th cells 14, including Th1, Th2 and Th17^15^-^17^. HDAC1 increases CD4^+^ T cell activation by inhibiting miR-124 expression and promoting IRF1 production in systemic lupus erythematosus 18. In classical Th17 cells, HDAC1 is recruited by growth factor independent 1 to repress CD73 expression, thereby inhibiting Th17 immunosuppressive properties 19.

Ubiquitination is an important protein modification that regulates diverse biological processes, including CD4^+^ T cell differentiation and function 20. The ubiquitination process is catalyzed by the sequential activation of the ubiquitin-activating (E1), ubiquitin-conjugating (E2), and ubiquitin-ligating (E3) enzymes 21. Different types of polyubiquitin chains are formed through the connection of the C-terminal glycine of ubiquitin to any of the seven internal lysine residues of the preceding ubiquitin 22. Lys48 (K48)-linked polyubiquitin chains mainly target proteins for proteasomal degradation, whereas K63-linked polyubiquitin chains mediate other functions, such as immune regulation 23. The RING finger (RNF) protein, which contains the RING domain, is the largest E3 ubiquitin ligase family with 340 validated human protein members 24, 25. Several RNF ubiquitin ligases are implicated in the regulation of CD4^+^ T cell differentiation and function 26-30. However, the function of most RNF ubiquitin ligases in CD4^+^ T cell differentiation and T cell-mediated pathological diseases remains unclear.

RNF157, which is one of the RNF ubiquitin ligases, plays a vital role in the regulation of neuronal survival and morphology 31, cell cycle 32, M2 macrophage polarization 33, and lens epithelial cell apoptosis 34. However, the specific role of RNF157 and its underlying mechanisms in CD4^+^ T-cell differentiation and MS pathogenesis remains unclear. In this study, we found that RNF157 expression was significantly decreased and showed a significant negative correlation with RORγt expression in MS. RNF157 attenuated EAE development through decreasing Th17 differentiation and expression of chemokine receptors, CCR4 and CXCR3. Mechanistically, RNF157 targeted HDAC1 for degradation through mediating K48-linked ubiquitination of HDAC1. These results suggest that RNF157 is a potential target in adaptive immune responses against MS and other autoimmune disorders.

## Materials and methods

### Mice

C57BL/6 mice (Wild type, WT) were from the Lab Animal Center of Southern Medicine University (Guangzhou, China).* Rnf157^flox/ flox^*(*Rnf157^fl/fl^*), and *Hdac1^fl/fl^* mice on a C57BL/6J background were generated by Cyagen Biosciences Inc. (Guangzhou, China) using CRISPR-Pro technology. CD4-Cre mice were from the Shanghai Research Center for Model Organisms (Shanghai, China). *Rnf157^fl/fl^*, or *Hdac1^fl/fl^* mice were crossed with CD4-Cre mice to generate* Rnf157^fl/fl^*; CD4-Cre (*Rnf157^CKO^*) mice, or *Hdac1^CKO^* mice. *Rnf157^CKO^* mice were crossed with* Hdac1^CKO^* mice to generate* Rnf157^CKO^Hdac1^CKO^* mice. CD45.1^+^ and *Rag1*^-/-^ mice were purchased from Nanjing Biomedical Research Institute (Nanjing, China). All mice were all C57BL/6 background and maintained in the Lab Animal Center of Southern Medicine University under specific pathogen-free conditions. All animal experiments were conducted in accordance with protocols approved by the Medical Ethics Board and the Biosafety Management Committee of Southern Medical University. All mice were used at an age of 6-12 weeks and were randomly divided into different groups.

### EAE model

C57BL/6 mice, *Rnf157^fl/fl^*, *Rnf157^CKO^*, *Hdac1^fl/fl^*, *Hdac1^CKO^*, *Rnf157^CKO^ Hdac1^CKO^* and recipient *Rag1*^-/-^ mice reconstituted by *Rnf157^fl/fl^* or *Rnf157^CKO^* CD4^+^ T cells were immunized subcutaneously with 200 μg MOG(35-55) peptide emulsified in CFA (Difco Laboratories, USA) with 400 μg *Mycobacterium tuberculosis* H37Ra on day 0. To induce EAE development and assess the severity of EAE, mice also received 200 ng of pertussis toxin (Sigma, USA) by intraperitoneal injection on days 0 and 2. Symptoms of EAE were monitored daily using a classical clinical score ranging from 0 to 5 as follows: 0, no disease; 1, tail paralysis; 2, weakness of hind limbs; 3, paralysis of hind limbs; 4, paralysis of hind limbs and severe hunched posture; 5, moribund or death, as previously described^35^.

### MOG(35-55) recall assay

Splenocytes or cells from central nervous system (Spinal cord and brain) were isolated from mice induced EAE, were re-stimulated with 100 μg/mL MOG(35-55) in complete RPMI1640 media for 6 h to perform flow cytometry analysis of intracellular IFN-γ, IL-4, or IL-17A; re-stimulated for 48 h to perform Enzyme-linked immunosorbent assay (ELISA).

### Mouse Naïve T cell isolation and T cell activation assay* in vitro*

Spleen and lymph node cells were isolated from mice. CD4^+^ T cells were negatively selected using EasySepTM. Mouse Naive CD4^+^ T cell Isolation Kit (Miltenyi, Germany). Purified naive T cells were stimulated with plate-bound anti-CD3 (1μg/mL or indicated concentrations) and soluble anti-CD28 antibodies (1μg/mL) in replicate wells of 96-well plates (1 × 10^5^ cells per well) for flow cytometry analysis and ELISA, 12-well plates (1 × 10^6^ cells per well) for qPCR and 6-well plates (5 × 10^6^ per well) for western blot assays.

### RNA-seq analyses

The Human RNA-seq data analyses were obtained from the NCBI GEO database, with accession number GSE66763^36^. Raw sequence data were downloaded and analyzed by using SRAdownload, HISAT2 (Ver. 2.0.5.2), and Cufflink (Ver. 2.2.1.0) tools from the usegalaxy.org. Heat maps of gene expression were generated using Heml soft.

### Human Naïve T cell isolation and T cell activation assay* in vitro*

Human Peripheral blood mononuclear cells were isolated from the peripheral blood of healthy donors by Ficoll centrifugation. Human Naïve CD4^+^ T cells were negatively selected using EasySepTM. Human Naive CD4^+^ T cell Isolation Kit (Miltenyi, Germany). Purified human naive T cells were stimulated with plate-bound anti-CD3 (1μg/mL or indicated concentrations) and soluble anti-CD28 antibodies (1μg/mL) in replicate wells of 96-well plates (1 × 10^5^ cells per well) for flow cytometry analysis and ELISA, 12-well plates (1 × 10^6^ cells per well) for qPCR and 6-well plates (5 × 10^6^ per well) for western blot assays. Informed consent was obtained in accordance with the Declaration of Helsinki and the Institutional Review Board of the Southern Medical University. Written informed consents were obtained from all participants for the use of PBMC samples.

### Flow cytometry analysis

For intracellular cytokine staining assays, T cells isolated from spleen or nervous system of mice, or from *in vitro* cultures were stimulated for 1.5 h with 100 mg/mL MOG(35-55) or PMA (50 ng/mL, Thermo Fisher Scientific, USA) and ionomycin (500 ng/mL, Thermo Fisher Scientific), before Brefeldin A (10 μg/mL, eBioscience, USA) was added to the culture for 3.5 h more. As previously described 37, for surface staining, cells were harvested, washed, and stained for 30 min on ice with mixtures of fluorescently conjugated mAbs or isotype-matched controls. For intracellular cytokine staining (ICS), cells were stained for surface molecules, fixed 20 min in IC Fixation buffer (Thermo Fisher Scientific), and incubated for 1 h in permeabilization buffer (Thermo Fisher Scientific) with appropriate mAbs of mice. Antibodies used in this study are listed in **Table S1**. Cell phenotype was analyzed by flow cytometry on a flow cytometer (BD LSR II) (BD Biosciences, USA) or Attune NxT (Thermo Fisher Scientific). Data were acquired as the fraction of labeled cells within a live-cell gate and analyzed using FlowJo software (Tree Star). All gates were set on the basis of isotype-matched control antibodies.

### CFSE T cell proliferation assay

Purified naïve T cells were labeled with 2.5 μM CFSE and then 5 × 10^4^ T cells/well were stimulated with anti-CD3 and anti-CD28. T cells were cultured for 72 h and proliferation was determined by flow cytometry analysis of CFSE dilution.

### CD4^+^ T cell differentiation

Purified human or mouse naive CD4^+^ T cells were stimulated with plate-bound anti-CD3 (2 μg/mL) and anti-CD28 (2 μg/mL) alone (Th0) or under Th1 (10 ng/mL IL-12 and 10 μg/mL anti-IL4, Peprotech, USA), Th2 (20 ng/mL IL-4 and 10 μg/mL anti-IFN-γ, Peprotech), Th17 (2.5 ng/mL TGF-β, 15 ng/mL IL-6, 10 μg/mL anti-IFN-γ and 10 μg/mL anti-IL4, Peprotech) and Treg (1.5 ng/mL TGF-β, 10 μg/mL anti-IFN-γ and 10 μg/mL anti-IL4, Peprotech) conditions. After 5 d of stimulation, the cells were subjected to Flow cytometry analysis, ELISA, or qPCR analyses.

### Enzyme-linked immunosorbent assay (ELISA)

Cytokine production in supernatants of *in vitro* cell cultures or sera of mice was measured by ELISA of mouse IFN-γ, IL-17, and IL-2 (ExCell Bio, China) according to the manufacturer's protocol.

### Quantitative PCR (qPCR) analysis

Total RNA was isolated with Trizol (Thermo Fisher Scientific) according to the manufacturer's instructions. 1 mg of RNA was reverse transcribed to cDNA with random RNA specific primers using the high-capacity cDNA reverse transcription kit (Applied Biosystems, USA). An Eppendorf Master Cycle Realplex2 and a SYBR Green PCR Master Mix (Applied Biosystems) were used for real-time PCR (40 cycles). The primer sequences used for PCR are in **Table S2**.

### Competitive T cell transfers

Spleens or lymph nodes were removed from naive wild-type (WT, CD45.1^+^) and *Rnf157^CKO^* (CD45.2^+^) mice, and CD4^+^ T cells were negatively selected using EasySepTM. 1×10^6^ WT CD45.1^+^ CD4^+^ T cells and 1×10^6^ *Rnf157^CKO^* CD45.2^+^ CD4^+^ T cells were mixed together and co-transferred into *Rag1*^-/-^ female mice via tail vein injection. One day later, the recipient mice were subjected to EAE induction.

### CD4^+^ T cell adoptive transfer

Spleens or lymph node were removed from naive *Rnf157^fl/fl^* and *Rnf157^CKO^* mice, and CD4^+^ T cells were negatively selected using EasySepTM. CD4^+^ T cells (1.0 × 106 per mouse) were injected via tail vein injection into *Rag1*^-/-^ female mice. One day later, the recipient mice were subjected to EAE induction.

### Plasmid constructs and transfection

Recombinant vectors encoding Flag-RNF157, Myc-HDAC1 (WT) or mutations (K74R, K89R or K361R) and HA-ub were cloned into the pcDNA3.1 (Sangon Biotech, China). The same or indicated quality plasmids were transfected into HEK293T cells with Lipofectamine 2000 (Invitrogen) according to manufacturer's instruction according to manufacturer's instruction.

### Immunoblot, co-immunoprecipitation and ubiquitination assays

The experiments were performed as previously described 38. Spinal cords or cells were washed three times with ice-cold PBS and then lysed in Nonidet P-40 lysis buffer containing 150 mM NaCl, 1mM EDTA, 1% Nonidet P-40, and 1% protease and phosphatase inhibitor cocktail (Biotool). Equal amounts (20 mg) of cell lysates were resolved using 8±15% polyacrylamide gels transferred to PVDF membrane. Membranes were blocked in 5% non-fat dry milk in PBST and incubated overnight with the respective primary antibodies at 4 ℃. The membranes were incubated at room temperature for 1 h with appropriate HRP-conjugated secondary antibodies and visualized with Plus-ECL (PerkinElmer, CA) according to the manufacturer's protocol. For immunoprecipitation assays, the lysates were immunoprecipitated with IgG or the appropriate antibodies and protein G Sepharose beads. The precipitates were washed three times with lysis buffer containing 500 mM NaCl, followed by immunoblot analysis. For deubiquitination assays, the cells were lysed with the lysis buffer and the supernatants were denatured at 95 °C for 5 min in the presence of 1% SDS. The denatured lysates were diluted with lysis buffer to reduce the concentration of SDS below 0.1% followed by immunoprecipitation with the indicated antibodies. The immunoprecipitates were subjected to immunoblot analysis with anti-ubiquitin chains. Antibodies used in this study are listed in **Supplementary Table 1**.

### Retroviral packaging and transduction

Genes encoding wild-type RNF157, or HDAC1 were cloned into retroviral vector pMXs containing IRES-regulated GFP (Youbio, China), respectively. Each of the resulting plasmids was transfected into a packaging cell line, PLAT-T, using FuGENE6 (Roche, Switzerland). After incubation for 24 h, the culture supernatant was harvested and condensed as a viral stock. The CD4^+^ T cells were stimulated by anti-CD3 and anti-CD28 antibodies for 24 h. The cells were then infected with retrovirus in the presence of 0.5 μg/mL of polybrene for 24 h and cultured further in the presence of 30 U/mL of IL-2 for 3 days. The cells were washed with fresh media and were stimulated with plate-bound anti-CD3 and anti-CD28 under Th17, or Treg conditions. After 5 d of stimulation, the cells were subjected to Flow cytometry analysis, ELISA, or qPCR analyses.

### Statistics

All experiments were performed at least thrice. When shown, multiple samples represent biological (not technical) replicates of mice randomly sorted into each experimental group. No blinding was performed during animal experiments. Determination of statistical differences was performed with Prism 8 (Graphpad Software, Inc.) using unpaired two-tailed *t*-tests (to compare two groups with similar variances).

## Results

### The potential critical role of RNF157 in autoimmunity

**Figure 1:**
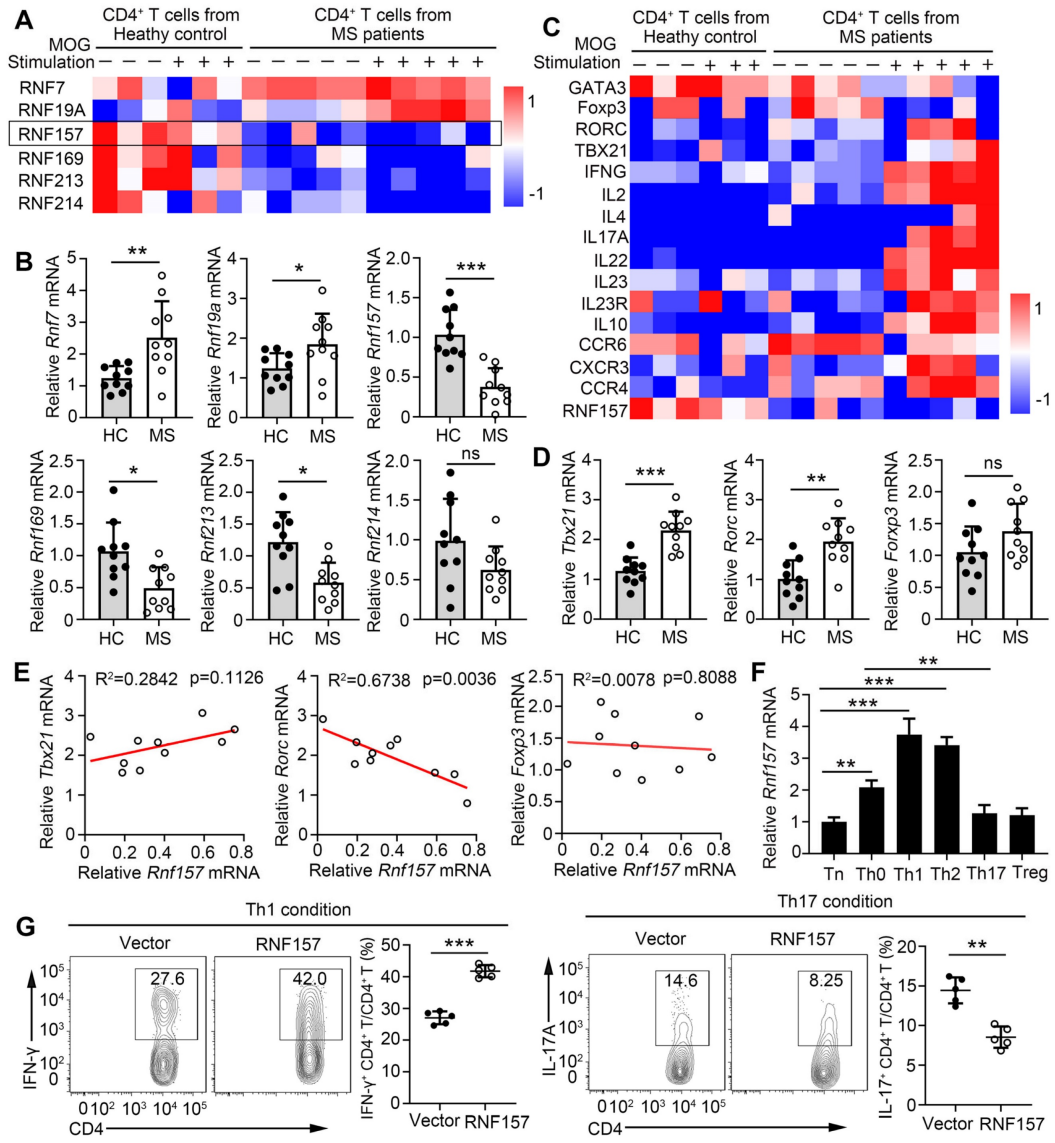
** RNF157 is associated with multiple sclerosis disease and involved in human CD4^+^ T cell differentiation. (A)** The heat map shows the relative expression of some RING-type E3 ubiquitin ligases from RNA-seq data comparing MOG-reactive or -non-reactive CD4^+^ T cells from multiple sclerosis subjects (MS) and healthy control individuals (HC).** (B)** RNF7, RNF19A, RNF157, RNF169, RNF213 and RNF214 mRNA expression were assessed using qPCR analysis in CD4^+^ T cells from MS and HC. **(C)** The heat map shows the relative amounts of RNA for MOG-reactive or -non-reactive CD4^+^ T cells from MS and HC. **(D)** T-bet, RORγt, and Foxp3 mRNA expression were assessed using qPCR analysis in CD4^+^ T cells from MS and HC. **(E)** The correlation of the expression of RNF157 with that of T-bet, RORγt, and Foxp3 in CD4^+^ T cells from HC (n = 10) and MS (n =10); results were plotted and analyzed with the linear-regression *t*-test. **(F)** Purified human naïve CD4^+^ T cells were isolated, and stimulated with anti-CD3 plus anti-CD28 (Th0), or under standard Th1, Th2, Th17 or Treg conditions, and harvested on day 5. RNF157 mRNA expression were assessed using qPCR. **(G)** Flow cytometry of intracellular IFN-γ or IL-17A in human naive CD4^+^ T cells infected with control retrovirus (Vector) or retrovirus expressing RNF157, and differentiated under standard Th1 conditions or Th17 conditions. Pooled data are presented in the below panel. Data shown are the mean ±SD. *P < 0.05, **P < 0.01 and ***P < 0.001 by an unpaired *t*-test. Data are representative of three independent experiments with similar results.

The direct contribution of several members of the E3 ubiquitin ligase family to CD4^+^ T cell function and MS development remains unclear. To identify the E3 ubiquitin ligases that participate in the pathogenesis of MS, we analyzed RNA sequencing (RNA-seq) data from the NCBI Gene Expression Omnibus. Gene expression was compared in MOG-non-reactive versus MOG-reactive CD4^+^ T cells isolated from healthy control individuals (HC) and patients with MS. The RNA-seq results showed that expression of E3 ubiquitin ligases, RNF7, RNF19A, RNF157, RNF169, RNF213, and RNF214, were significantly different between the CD4^+^ T cells of the HC and patients with MS (Figure 1A). Subsequently, the sequencing results were verified via qPCR and showed the most significant difference in the expression of RNF157 (Figure 1B). CD4^+^ T cells, especially MOG-specific CD4^+^ T cells, from patients with MS expressed low amounts of RNF157 and showed elevated levels of TBX21 (T-bet), RORC (RORγt), IFNG, and IL17A, compared with CD4^+^ T cells from HC (Figure 1C). qPCR results also showed that TBX21 and RORC expression were increased in CD4^+^ T cells from patients with MS compared with those in CD4^+^ T cells from HC (Figure 1D). The expression of RORC and RNF157 showed a significant negative correlation in CD4^+^ T cells from patients with MS (Figure 1E). However, TBX21 and RNF157 exhibited coexpression; however, it was not significant (Figure 1E).

Relative to its expression in Th0 cells, higher RNF157 expression was observed in human Th1 and Th2 cells, whereas lower RNF157 expression was observed in Th17 and Treg cells (Figure 1F). RNF157 overexpression in human naive CD4^+^ T cells promoted intracellular expression and secretion of IFN-γ after stimulation with anti-CD3 and CD28 (Figure S1A and B). However, it did not affect IL-2 secretion (Supplementary Fig. 1B). Activation analysis showed that RNF157 overexpression did not affect CD69, CD44, and CD62 levels (Figure S1C). No differences in proliferation were observed between CD4^+^ T cells with or without RNF157 overexpression (Figure S1D). Notably, RNF157 overexpression in human naive CD4^+^ T cells promoted Th1 and Th2 differentiation, attenuated Th17 differentiation, and did not affect Treg differentiation (Figure 1G and Figure S1E). Overall, these results indicated that RNF157 may serve a critical role in autoimmunity through regulating the differentiation of CD4^+^ T cell subpopulations.

### RNF157 regulated *in vivo* CD4^+^ T cell differentiation during autoimmunity

**Figure 2:**
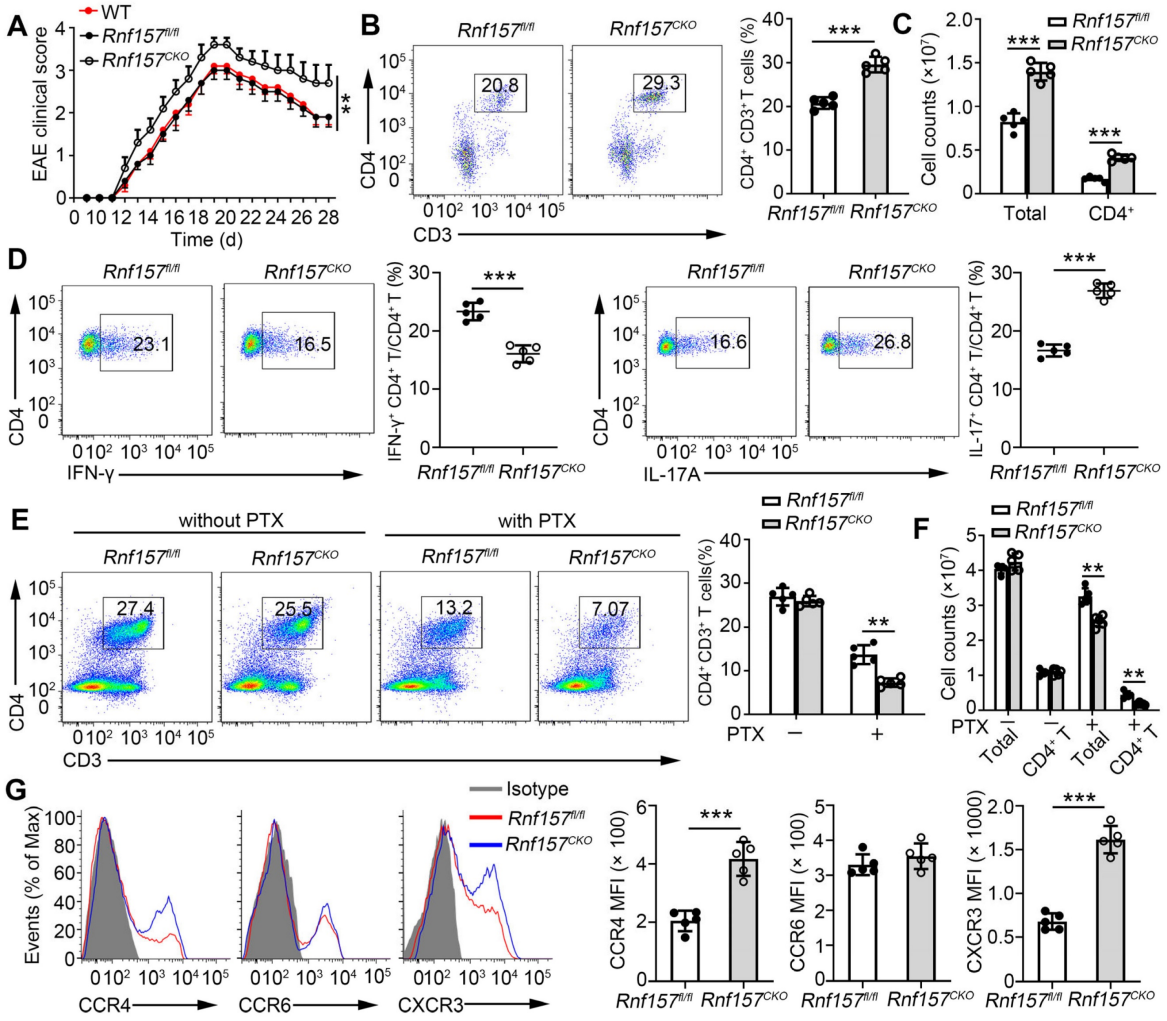
** RNF157 deficiency in CD4^+^ T cells promoted EAE development through regulating CD4^+^ T cell responses and chemotaxis to the central nervous system. (A-D)** WT,* Rnf157^fl/fl^* and *Rnf157^CKO^* mice were immunized with MOG(35-55) peptide in CFA adjuvant and pertussis toxin (PTX) to induce EAE. **(A)** The graph shows the clinical score of EAE (n = 10 for *Rnf157^fl/fl^* and *Rnf157^CKO^* mice respectively).** (B)** Percentage of CD4^+^ T cells among cells infiltrating to the central nervous system was analyzed by flow cytometry and pooled data are presented in the right panel. **(C)** Total number of cells and CD4^+^ T cells infiltrating the central nervous system. **(D)** The cells from the central nervous system (the spinal cord and brain) were restimulated directly ex vivo and the intracellular production of IFN-γ and IL-17A by CD4^+^ T cells was determined. Pooled data are presented in the right panel. **(E-G)*** Rnf157^fl/fl^* and *Rnf157^CKO^* mice were immunized with MOG(35-55) peptide in CFA adjuvant, and the mice were then treated with **(E-F)** or without **(E-G)** PTX. **(E)** Percentage of CD4^+^ T cells in spleen was analyzed by flow cytometry and pooled data are presented in the right panel.** (F)** Total number of cells and CD4^+^ T cells in spleens. **(G)** Representative flow cytometry data showing CCR4, CCR6, and CXCR3 on CD4^+^ T cells from spleens of *Rnf157^fl/fl^* and *Rnf157^CKO^* mice. Pooled data of mean fluorescence intensity (MFI) are presented in the right panel. Data shown are the mean ±SD. *P < 0.05, **P < 0.01 and ***P < 0.001 by an unpaired *t*-test. Data are representative of three independent experiments with similar results.

**Figure 3:**
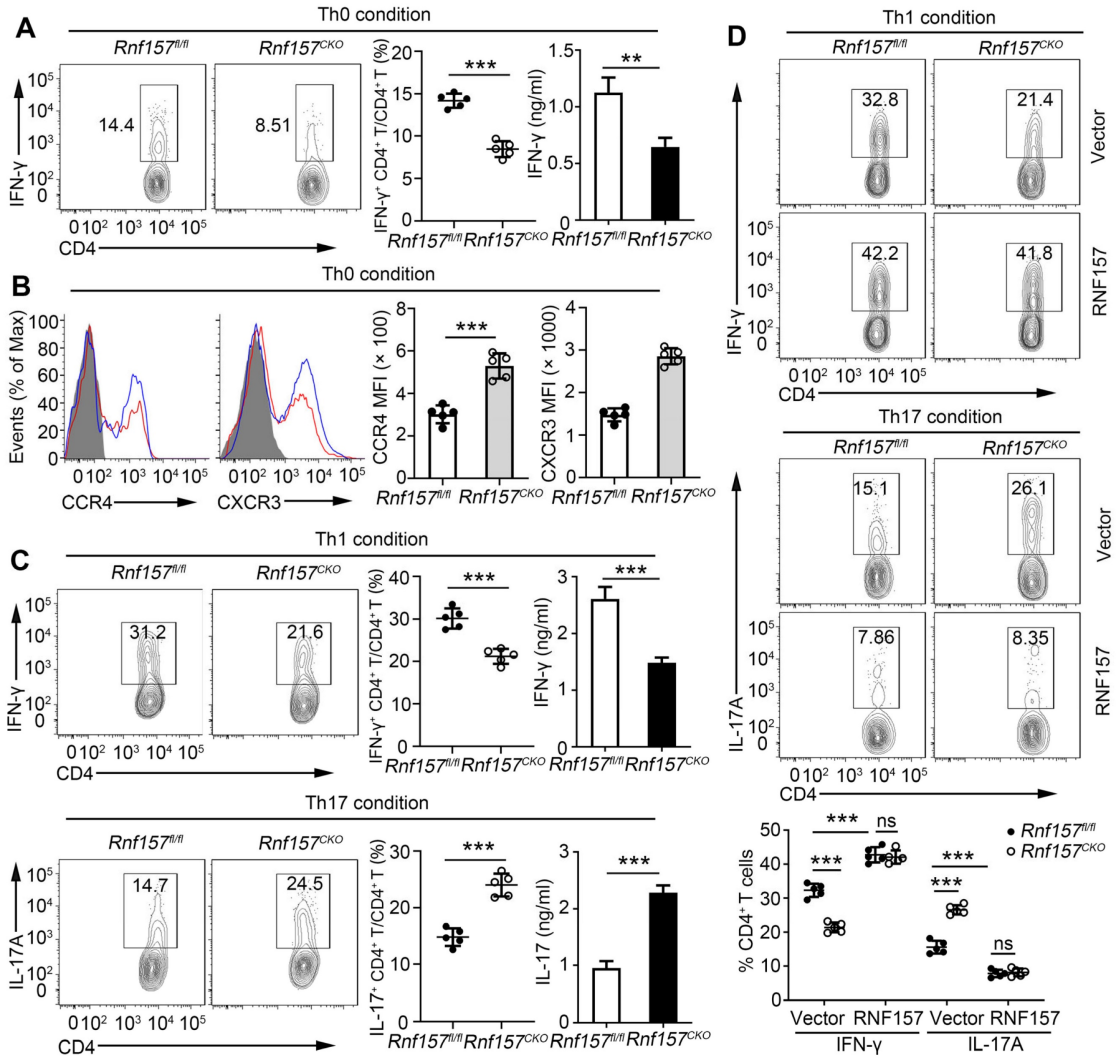
** RNF157 deficiency attenuated Th1 cell differentiation but promoted Th17 cell differentiation and expression of CCR4 and CXCR3 on CD4^+^ T cells. (A-C)** Purified naïve CD4^+^ T cells from *Rnf157^fl/fl^* and *Rnf157^CKO^* mice were isolated, and stimulated with anti-CD3 plus anti-CD28 (Th0 condition), or under standard Th1 or Th17 condition, and harvested on day 5. **(A)** Flow cytometry of intracellular IFN-γ and pooled data (near right) in CD4^+^ T cells. ELISA results of IFN-γ in the culture medium are respectively presented in the far right panels. **(B)** Representative flow cytometry data showing CCR4 and CXCR3 on CD4^+^ T cells. Pooled data of mean fluorescence intensity (MFI) are presented in the right panel. **(C)** Flow cytometry of intracellular IFN-γ or IL-17A and pooled data (near right) in CD4^+^ T cells. ELISA results of IFN-γ or IL-17A in the culture medium are respectively presented in the far right panels. **(D)** Flow cytometry of intracellular IFN-γ or IL-17A in *Rnf157^fl/fl^* and *Rnf157^CKO^* naive CD4^+^ T cells infected with control retrovirus (Vector) or retrovirus expressing RNF157 and differentiated under standard Th1 conditions or Th17 conditions. Pooled data are presented in the below panel. Data shown are the mean ±SD. *P < 0.05, **P < 0.01 and ***P < 0.001 by an unpaired *t*-test. Data are representative of three independent experiments with similar results.

To further analyze the function of RNF157 in CD4^+^ T cells, we generated mice with conditional RNF157 knockout in CD4^+^ T cells (*Rnf157^CKO^*) through crossing *Rnf157^flox/flox^* (*Rnf157^fl/fl^*) and CD4-Cre mice. *Rnf157^CKO^* mice did not show any distinct abnormalities in thymocyte development or peripheral T cell homeostasis (Figure S2A-E), including intracellular IFN-γ and IL-17A expression in splenic CD4^+^ T cells (Figure S2F). To explore the role of RNF157 in CD4^+^ T cell differentiation and autoimmunity, *Rnf157^fl/fl^* and *Rnf157^CKO^* mice were immunized with MOG(35-55) peptide in complete Freund's adjuvant (CFA) and treated with pertussis toxin (PTX) to induce EAE. The results showed that RNF157 deficiency in CD4^+^ T cells promoted EAE progression (Figure 2A). Compared with *Rnf157^fl/fl^* mice, *Rnf157^CKO^* mice produced lower serum and spinal cord levels of IFN-γ and higher serum and spinal cord levels of IL-17A (Figure S3A). There were no differences in serum IL-2 concentration between *Rnf157^fl/fl^* and *Rnf157^CKO^* mice (Figure S3A). Flow cytometry results showed that the percentage of CD4^+^ T cells infiltrating the CNS was significantly higher in *Rnf157^CKO^* mice (Figure 2B). The amount of total infiltrating and CD4^+^ T cells were also higher in the CNS of *Rnf157^CKO^* mice (Figure 2C). Notably, after MOG(35-55) peptide immunization, CD4^+^ T cells in the CNS of *Rnf157^CKO^* mice showed decreased intracellular expression of IFN-γ and IL-4 and increased intracellular expression of IL-17A compared with those of *Rnf157^fl/fl^* mice (Figure 2D and FigureS 3B). The proportion of Treg cells (CD25^+^ Foxp3^+^) among CD4^+^ T cells was not affected by RNF157 deficiency (Figure S3C). Neutrophils represented an increased fraction of the infiltrating myeloid cell population in *Rnf157^CKO^* mice (Figure S3D). Moreover, RNF157 did not affect cell apoptosis (Figure S3E). The proportion of CD4^+^ T cells in the spleens showed no significant difference between *Rnf157^fl/fl^* and *Rnf157^CKO^* mice treated without PTX (as the cells could not infiltrate CNS); however, with PTX treatment, the proportion of CD4^+^ T cells in the spleens of *Rnf157^CKO^* mice was lower compared with that in the spleens of *Rnf157^fl/fl^* mice (Figure 2E). The amount of total and CD4^+^ T cells in the spleens of *Rnf157^CKO^* mice also decreased (Figure 2F). In EAE, chemokine receptors play an important role in T cell chemotaxis to the CNS. Previous RNA-seq results showed that CCR4, CCR6, and CXCR3 in MOG-specific CD4^+^ T cells (Figure 1C). We also found that RNF157 deficiency promoted the expression of CCR4 and CXCR3 but not CCR6 in the splenic CD4^+^ T cells from *Rnf157^fl/fl^* and *Rnf157^CKO^* mice treated without PTX (Figure 2G). Overall, these results suggested that RNF157 deficiency in CD4^+^ T cells promotes EAE development by regulating the differentiation of CD4^+^ T cells and expression of chemokine receptors, CCR4 and CXCR3.

### RNF157 regulated *in vitro* differentiation of CD4^+^ T cells and expression of CCR4 and CXCR3

We further determined the role of RNF157 in regulating CD4^+^ T cells *in vitro*. The expression trend of RNF157 was similar in different subtypes of mouse and human CD4^+^ T cells (Figure S4A). The expression of the activation markers, CD69, CD44, and CD62L, production of the cytokine, IL-2, and cell proliferation was not affected by the deletion of RNF157 following treatment with anti-CD3 and anti-CD28 antibodies (Figure S4B-D). Intracellular expression and secretion of IFN-γ were decreased in *Rnf157^CKO^* CD4^+^ T cells (Figure 3A). However, *Rnf157^CKO^* CD4^+^ T cells expressed higher levels of CCR4 and CXCR3 than did *Rnf157^fl/fl^* CD4^+^ T cells (Figure 3B). CCR6 expression showed no difference between *Rnf157^fl/fl^* and *Rnf157^CKO^* CD4^+^ T cells (Figure SFig. 4E). Under standard differentiation conditions, RNF157 deficiency in CD4^+^ T cells decreased Th1 and Th2 differentiation, promoted Th17 differentiation, and had no effect on Treg differentiation (Figure 3C and Figure S4F). Furthermore, reintroduction of RNF157 into *Rnf157^CKO^* CD4^+^ T cells rescued Th1 differentiation and attenuated Th17 differentiation (Figure 3D). These results suggested that RNF157 regulates the *in vitro* differentiation of CD4^+^ T cells and expressions of CCR4 and CXCR3.

### RNF157 regulated CD4^+^ T cell functions in a T cell-intrinsic manner *in vivo*

**Figure 4:**
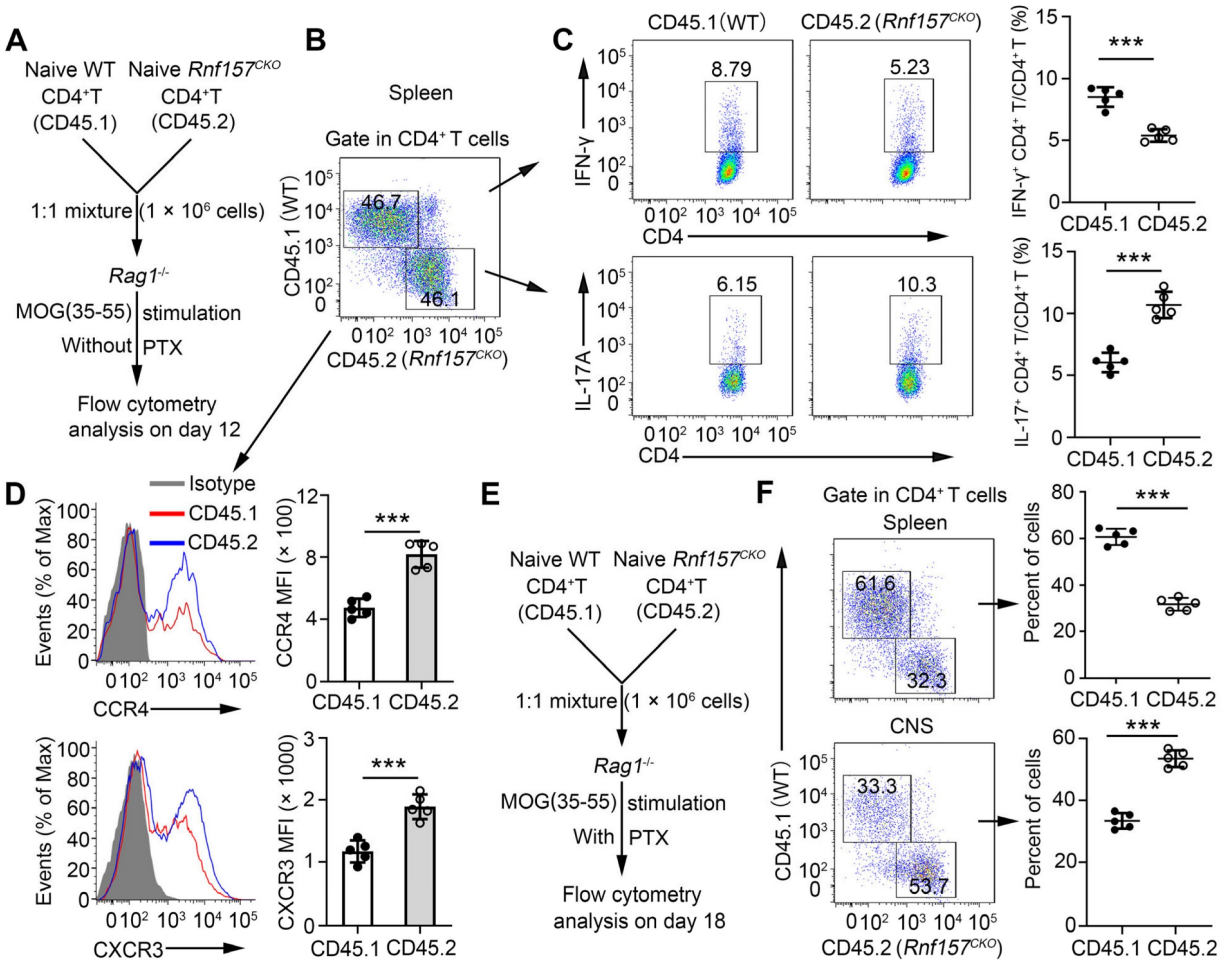
** RNF157 regulated CD4^+^ T cell differentiation and expression of CCR4 and CXCR3 in a T-cell-intrinsic manner *in vivo*. (A)** Schematic of experimental design of competitive adoptive CD4^+^ T cell transfer assays for **(B-D)**. **(B)** The percentages of wild-type (WT, CD45.1^+^) and and *Rnf157^CKO^* (CD45.2^+^) in the CD4^+^ T cell populations from spleens was determined. **(C)** Splenocytes were restimulated directly ex vivo and the intracellular production of IFN-γ and IL-17 by CD45.1^+^ or CD45.2^+^ CD4^+^ T cells was determined. Pooled data are presented in the right panel. **(D)** Representative flow cytometry data showing CCR4 and CXCR3 on CD4^+^ T cells. Pooled data of mean fluorescence intensity (MFI) are presented in the right panel. **(E)** Schematic of experimental design of competitive adoptive CD4^+^ T cell transfer assays for **(F)**. **(F)** The percentages of CD45.1^+^ and CD45.2^+^ in the CD4^+^ T cell populations from spleens and central nervous system was determined. Data shown are the mean ±SD. *P < 0.05, **P < 0.01 and ***P < 0.001 by an unpaired *t*-test. Data are representative of three independent experiments with similar results.

We conducted *in vivo* competitive adoptive CD4^+^ T cell transfer assays to confirm the intrinsic role of RNF157 in regulating CD4^+^ T cell functions. *Rag1*^-/-^ recipient mice received CD45.1^+^ wild-type (WT) and CD45.2^+^ *Rnf157^CKO^* naive CD4^+^ T cells (1:1) and were subsequently immunized with MOG(35-55) in CFA (Figure 4A). CD4^+^ T cell percentages of CD45.1^+^ WT and CD45.2^+^ *Rnf157^CKO^* cells showed no significant difference (Figure 4B). However, CD45.2^+^ *Rnf157^CKO^* CD4^+^ T cells exhibited considerably lower percentage of Th1 cells, higher proportion of Th17 cells (Figure 4C), and increased expressions of CCR4 and CXCR3 compared with CD45.1^+^ WT CD4^+^ T cells (Figure 4D). Furthermore, PTX treatment of *Rag1*^-/-^ recipient mice effected significantly higher and lower percentages of CD45.2^+^ *Rnf157^CKO^* CD4^+^ T cells than those of CD45.1^+^ WT CD4^+^ T cells in the spleen and CNS, respectively (Figure 4E-F).

To further analyze the effect of *Rnf157^CKO^* CD4^+^ T cells on the *in vivo* immune response, *Rag1*^-/-^ recipient mice were administered with *Rnf157^fl/fl^* and *Rnf157^CKO^* naive CD4^+^ T cells and subsequently immunized with MOG(35-55) peptide in CFA and PTX to induce EAE. *Rag1*^-/-^ mice with *Rnf157^CKO^* CD4^+^ T cells developed significantly enhanced EAE (Figure S5A), showed lower concentration of IFN-γ, and higher concentration of IL-17A (Figure S5B) than those with *Rnf157^fl/fl^* CD4^+^ T cells. The percentage and amount of CD4^+^ T cells infiltrating the CNS were increased in *Rag1*^-/-^ mice with* Rnf157^CKO^* CD4^+^ T cells (Figure S5C-D). In addition, after stimulation with the MOG(35-55) peptide, *Rnf157^CKO^* CD4^+^ T cells exhibited significantly lower percentage of Th1 and higher percentage of Th17 cells than *Rnf157^fl/fl^* CD4^+^ T cells (Figure S5E). Therefore, these results further demonstrated that RNF157 regulates CD4^+^ T cell functions in a T cell-intrinsic manner.

### RNF157 promoted HDAC1 degradation in CD4^+^ T cells

**Figure 5:**
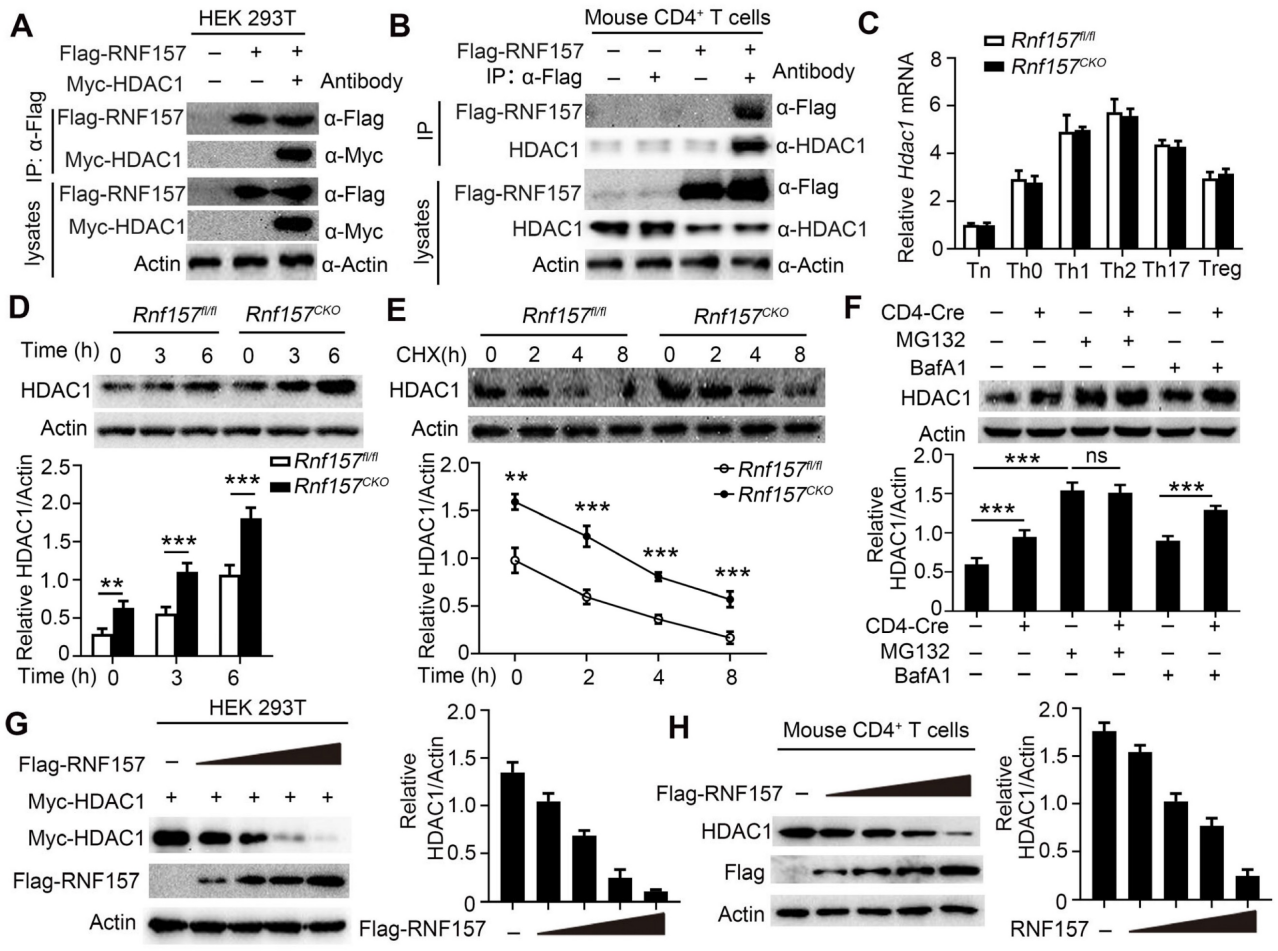
** RNF157 promoted the degradation of HDAC1 in CD4^+^ T cells. (A)** Immunoprecipitation (IP) and immunoblot (IB) analysis of HEK293 cells that were transfected with indicated plasmids for 24 h. **(B)** Purified naïve CD4^+^ T cells from WT mice were isolated, infected with control retrovirus (-) or retrovirus exrepssiong Flag-RNF157 (+), and stimulated with anti-CD3 plus anti-CD28 for 12 h. IP and IB analysis were then carried out. **(C)** Purified naïve CD4^+^ T cells from *Rnf157^fl/fl^* and *Rnf157^CKO^* mice were stimulated under standard Th0, Th1, Th2, Th17 or Treg conditions and harvested on day 5. HDAC1 expression levels were detected by qPCR.** (D-F)** HDAC1 IB analysis using whole-cell extracts of *Rnf157^fl/fl^* and *Rnf157^CKO^* CD4^+^ T cells stimulated with anti-CD3 and anti-CD28 for indicated time **(D-E)** or 3 h **(F)**. Cycloheximide (CHX), MG132 or BafA1 was added in indicated assays. Densitometry quantification of band intensity are respectively presented in the below panel.** (G)** IB analysis of HEK293T cells transfected with Myc-HDAC1 and increasing doses of expression vector for Flag-RNF157 (wedge). Densitometry quantification of band intensity is presented in the right panel. **(H)** Purified naïve CD4^+^ T cells from WT mice were isolated, infected with control retrovirus (-) or increasing doses of retrovirus exrepssiong Flag-RNF157 (wedge), and then stimulated with anti-CD3 and anti-CD28 for 3 h. HDAC1 IB analysis were then carried out. Data shown are the mean ±SD. *P < 0.05, **P < 0.01 and ***P < 0.001 by an unpaired *t*-test. Data are representative of three independent experiments with similar results.

We explored the molecular mechanisms underlying RNF157 in CD4^+^ T cell differentiation. First, the related proteins that may bind human and mouse RNF157 were predicted using BioGRID (Auto, https://thebiogrid.org/), HitPredict (Auto, http://www.hitpredict.org/), and STRING (minimum required interaction score > 0.200, https://version11.string-db.org/). Three methods simultaneously identified eight proteins that potentially bind human RNF157 (Figure S6A). HDAC1 has been shown as a key regulator of CD4^+^ T cell-mediated immunity in mice and human CD4^+^ T cells 13. However, the other eight proteins were less documented in the regulation of CD4^+^ T cell functions. Thus, we investigated whether RNF157 regulates HDAC1 in CD4^+^ T cells. We found that endogenous RNF157 formed a stable complex with HDAC1 in human CD4^+^ T cells (Figure S6B). In addition, mouse RNF157 interacted with HDAC1 in HEK293 cells (Figure 5A). Because of unavailability of mouse RNF157 antibody, we first overexpressed Flag-RNF157 in mouse CD4^+^ T cells and performed co-IP using Flag antibody. The co-IP results showed that mouse RNF157 also bound HDAC1 (Figure 5B). HDAC1 expression between *Rnf157^fl/fl^* and *Rnf157^CKO^* CD4^+^ T cells did not differ at the mRNA level (Figure 5C) but showed a significant difference at the protein level (Figure 5D). QPCR results also showed that HDAC1 mRNA expression was not different in CD4^+^ T cells from patients with MS compared with those in CD4^+^ T cells from HC (Figure S6C). The expression of HDAC1 and RNF157 did not show a significant correlation in CD4^+^ T cells from patients with MS (Figure S6C). RNF157 deficiency significantly attenuated the degradation of HDAC1 (Figure 5E), and the HDAC1 degradation was completely blocked by the proteasome inhibitor, MG132, but not by the autophagy inhibitor, BafA1 (Figure 5F). We further examined whether RNF157 downregulates HDAC1. The results showed that RNF157 downregulated HDAC1 in HEK293T cells (Figure 5G) and mouse (Figure 5H) and human CD4^+^ T cells (Figure S6D) in a dose-dependent manner. Among the functional domains of the RNF proteins, the RING domain facilitates the transfer of ubiquitin from the E2 to the substrate 39. Our findings indicate that the absence of the RING domain (RNF157 ΔRING) resulted in the inability of RNF157 to degrade HDAC1 (Figure S6E). These results demonstrated that RNF157 targets HDAC1 and promotes HDAC1 degradation in CD4^+^ T cells.

### RNF157 mediated K48-linked ubiquitination of HDAC1

**Figure 6:**
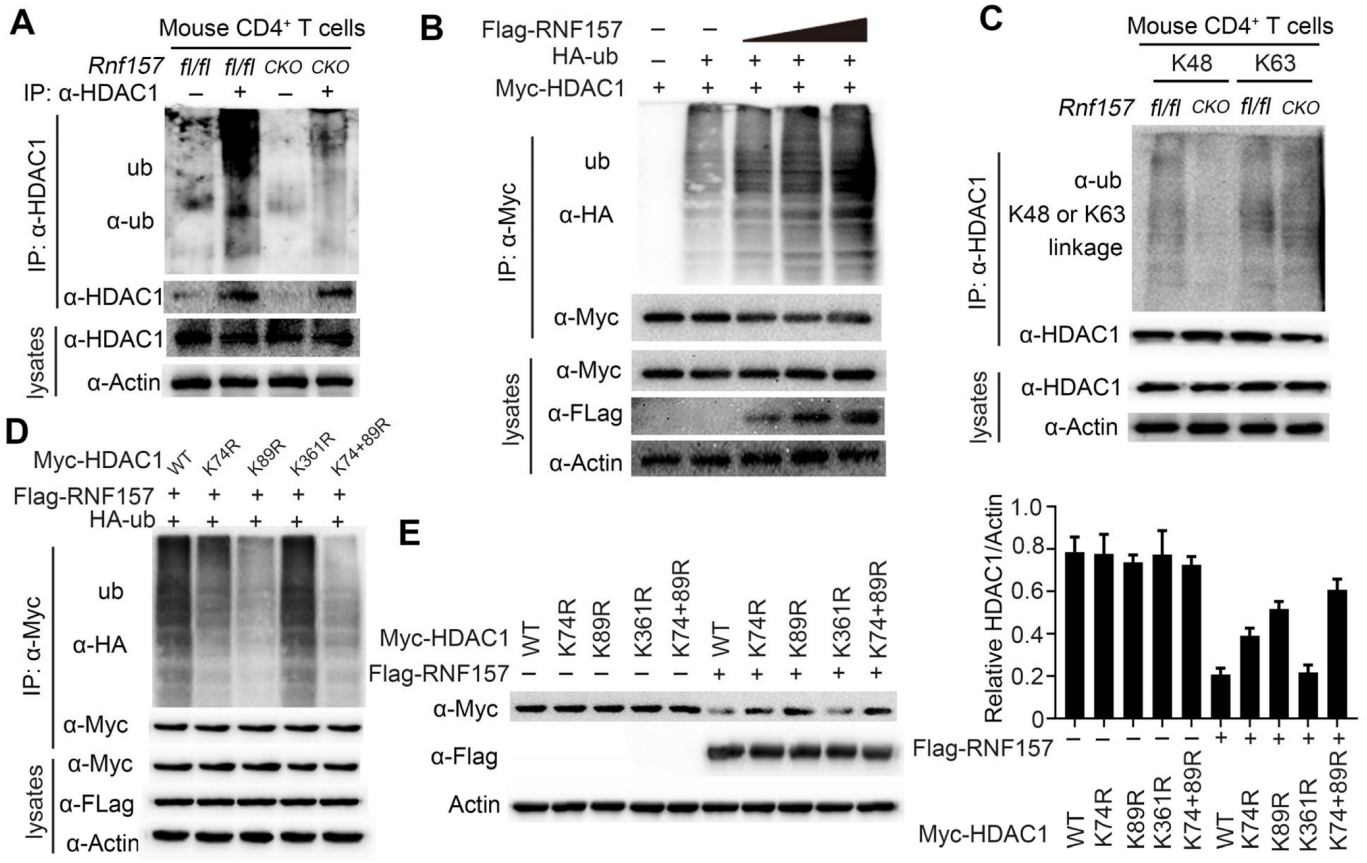
** RNF157 ubiquitinates HDAC1 in CD4^+^ T cells. (A)** Denature- Immunoprecipitation (IP) and immunoblot (IB) analysis of *Rnf157^fl/fl^* and *Rnf157^CKO^* CD4^+^ T cells stimulated with anti-CD3 and anti-CD28 for 3 h. MG132 was added to inhabit ubiquitin-proteasome.** (B)** Denature-IP and IB analysis of HEK 293T cells transfected with Myc-HDAC1, HA- ubiquitin (ub), and increasing doses of expression vector for Flag-RNF157 (wedge). MG132 was added to inhabit ubiquitin-proteasome. **(C)** Denature- Immunoprecipitation (IP) and immunoblot (IB) analysis (with anti-K48 or K63 linkage polyubiquitin) of *Rnf157^fl/fl^* and *Rnf157^CKO^* CD4^+^ T cells stimulated with anti-CD3 and anti-CD28 for 3 h. MG132 was added to inhabit ubiquitin-proteasome.** (D)** Denature-IP and IB analysis of HEK 293T cells transfected with Myc-HDAC1 (WT) or mutations (K74R, K89R, K361R, or K74 /89R), HA- ubiquitin (ub), and Flag-RNF157. MG132 was added to inhabit ubiquitin-proteasome.** (E)** IB analysis of HEK 293T cells transfected with Myc-HDAC1 (WT) or mutations (K74R, K89R, K361R, or K74 /89R), HA- ubiquitin (ub), and Flag-RNF157. Densitometry quantification of Myc-HDAC1 band intensity is presented in the right panel. Data shown are the mean ±SD. *P < 0.05, **P < 0.01 and ***P < 0.001 by an unpaired *t*-test. Data are representative of three independent experiments with similar results.

As RNF157 interacted with HDAC1 and promoted its degradation in CD4^+^ T cells, we investigated whether RNF157, which is an E3 ubiquitin ligase, functions through conjugating polyubiquitin chains to HDAC1. Consistently, RNF157 deficiency decreased the ubiquitination level of HDAC1 in CD4^+^ T cells (Figure 6A). By contrast, overexpression of RNF157 increased the ubiquitination level of HDAC1 in HEK293T cells in a dose-dependent manner (Figure 6B). To examine the type of RNF157-mediated ubiquitin linkage of HDAC1, K48 or K63-linked specific polyubiquitin antibody was used. The results showed that K48-linked ubiquitination of HDAC1 was significantly decreased in RNF157-deficient CD4^+^ T cells (Figure 6C). Furthermore, K74, K89, and K361 were predicted as the E3-specific ubiquitination sites of HDAC1 according to the Protein Lysine Modifications Database (http://plmd.biocuckoo.org/). However, site mapping analysis revealed that mutation of K74 or K89 into arginine residues impaired the RNF157-mediated ubiquitination of HDAC1 (Figure 6D). Similarly, mutation of K74 or K89 into arginine residues impaired RNF157-mediated degradation of HDAC1 (Figure 6E). Overall, these results demonstrated that RNF157 promotes the degradation of HDAC1 through mediating K48-linked ubiquitination of HDAC1 in CD4^+^ T cells.

### RNF157-regulated CD4^+^ T cell functions depend on HDAC1

**Figure 7:**
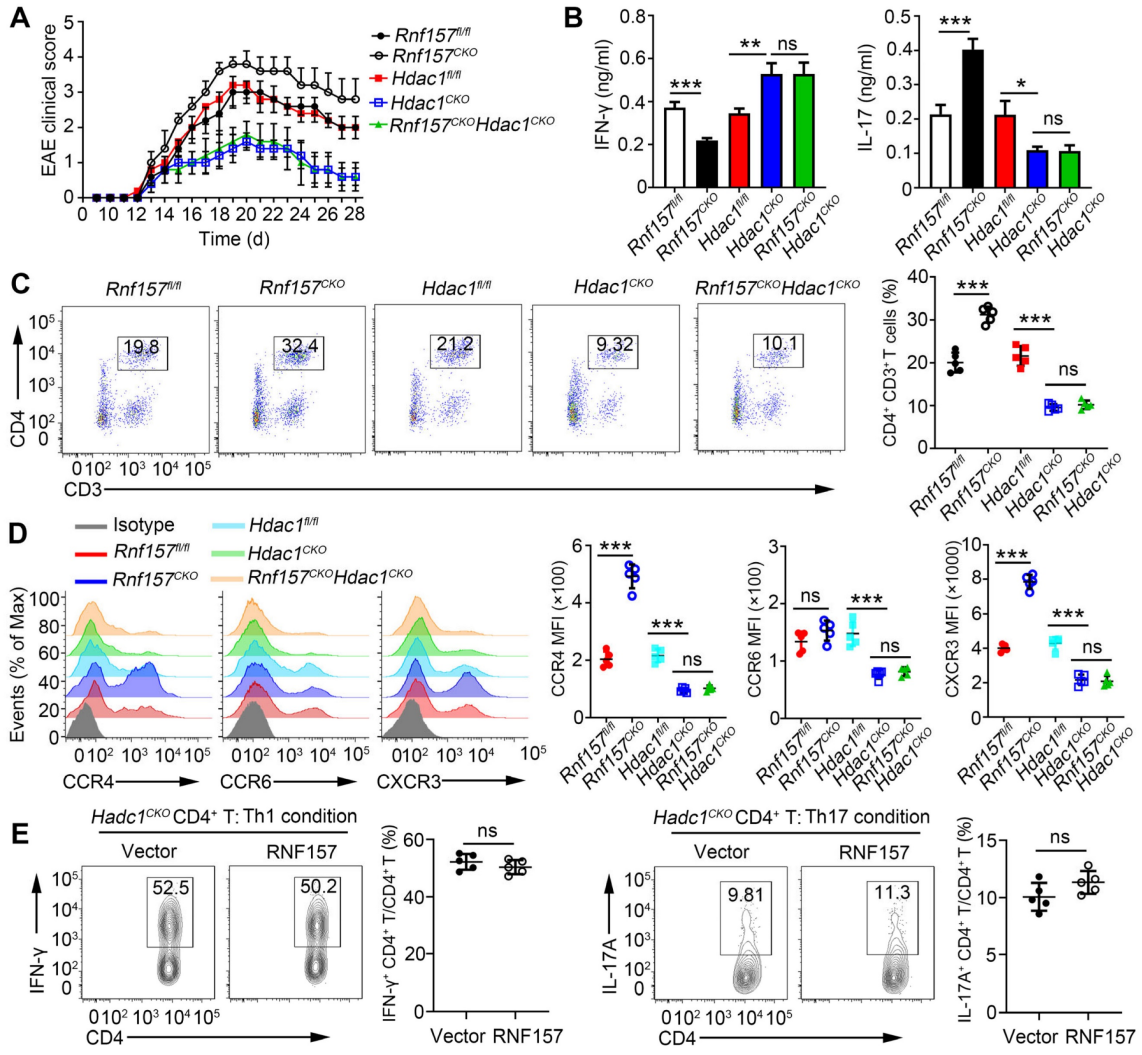
** RNF157 regulated CD4^+^ T cell differentiation and expression of CCR4 and CXCR3 dependent on HDAC1. (A-C)** *Rnf157^fl/fl^*, *Rnf157^CKO^*, *Hdac1^fl/fl^*, *Hdac1^CKO^* and *Rnf157^CKO^ Hdac1^CKO^* mice were immunized with MOG(35-55) peptide in CFA adjuvant and pertussis toxin (PTX) to induce EAE. **(A)** The graph shows the clinical score of EAE (n = 5 respectively). **(B)** Mice were harvested on day 28 and concentration of IFN-γ and IL-17 in serum was measured by ELISA. **(C)** Percentage of CD4^+^ T cells among cells infiltrating to the central nervous system was analyzed by flow cytometry and pooled data are presented in the right panel. **(D)** *Rnf157^fl/fl^*, *Rnf157^CKO^*, *Hdac1^fl/fl^*, *Hdac1^CKO^* and *Rnf157^CKO^ Hdac1^CKO^* mice were immunized with MOG(35-55) peptide in CFA adjuvant and these mice were harvested on day 12. Representative flow cytometry data showing CCR4, CCR6 and CXCR3 on CD4^+^ T cells. Pooled data of mean fluorescence intensity (MFI) are presented in the right panel. **(E)** Flow cytometry and of intracellular IFN-γ or IL-17A in *Hdac1^CKO^* naive CD4^+^ T cells infected with control retrovirus (Vector) or retrovirus expressing RNF157 and differentiated under standard Th1 conditions or Th17 conditions. Data shown are the mean ±SD. *P < 0.05, **P < 0.01 and ***P < 0.001 by an unpaired *t*-test. Data are representative of three independent experiments with similar results.

To assess whether HDAC1 is the primary target of RNF157 in regulating CD4^+^ T cell function, mice double conditional knockout for RNF157 and HDAC1 in CD4^+^ T cells (*Rnf157^CKO^Hdac1^CKO^*) were generated. *Rnf157^fl/fl^*, *Rnf157^CKO^*,* Hdac1^fl/fl^*, *Hdac1^CKO^,* and *Rnf157^CKO^ Hdac1^CKO^* mice were then immunized with MOG(35-55) peptide and PTX to induce EAE. The results showed that conditional knockout of HDAC1 in CD4^+^ T cells completely abolished the effect of RNF157 on EAE scores (Figure 7A) and serum levels of IFN-γ and IL-17A (Figure 7B). The percentage of CD4^+^ T cells among cells infiltrating the CNS was significantly increased in *Rnf157^CKO^* mice compared with that in *Rnf157^fl/fl^* mice. However, this difference was not observed when mice were also deficient for HDAC1 (Figure 7C). Similarly, the difference in intracellular IFN-γ and IL-17 levels and CCR4 and CXCR3 expression between *Rnf157^fl/fl^* and *Rnf157^CKO^* CD4^+^ T cells were not observed when HDAC1 was deficient (Figure S7A-B, and Figure 7D). Notably, overexpression of RNF157 in *Hdac1^CKO^* naive T cells had no effect on the differentiation of Th1 or Th17 cells (Figure 7E). HDAC1 overexpression in *Rnf157^CKO^* naive CD4^+^ T cells attenuated the generation of Th1 cells, but completely rescued the generation of Th17 cells (Figure S7C). These results demonstrated that RNF157 regulates CD4^+^ T cell function in an HDAC1-dependent manner.

## Discussion

CD4^+^ T cells play an important role in body development and homeostasis; thus, their differentiation and function are tightly regulated under normal conditions. Quantitative and functional changes in CD4^+^ T cells result in abnormal immune responses, which lead to inflammation, cancer, or autoimmune diseases 40. An increasing number of studies have reported that RNF ubiquitin ligases play a vital role in regulating functions of CD4^+^ T cells. Studies have reported that RNF ubiquitin ligases play a vital role in regulating CD4^+^ T cell functions. RNF128 (also called GRAIL) induces the anergic phenotype of CD4^+^ T cells through ubiquitin-mediated regulation of proteins essential for mitogenic cytokine expression 41, and regulates primary CD4^+^ T cell activation, survival, and differentiation 42. RNF56- (also called Cbl-b) deficient mice show impaired TGF-β-induced Foxp3 expression 29. RNF31 positively regulates Treg cell function through stabilizing FOXP3^26^. In this study, we identified RNF157 as a vital regulator of CD4^+^ T cell differentiation and function. In CD4^+^ T cells, RNF157 targeted HDAC1 for K48-linked ubiquitination and degradation. Therefore, targeting these RNF ubiquitin ligases may regulate CD4^+^ T cell differentiation and function and limit the occurrence and development of related diseases.

RNF157 is a novel E3 ubiquitin ligase and is demonstrated to play an important regulatory role in cell cycle and apoptosis. RNF157 in mammalian neurons regulates dendrite growth and neuronal survival, and the adaptor protein, APBB1, is an interactor and proteolytic substrate of RNF157^31^. Knockdown of endogenous RNF157 in melanoma cells leads to late S phase and G(2)/M arrest and induces apoptosis. Moreover, RNF157 inhibits lens epithelial cell apoptosis through negatively regulating p53 in age-related cataracts 34. However, no differences in proliferation were observed between human CD4^+^ T cells with or without RNF157 overexpression, as well as between *Rnf157^fl/fl^* and *Rnf157^CKO^* CD4^+^ T cells. Furthermore, RNF157 deficiency in CD4^+^ T cells did not affect cell apoptosis. RNF157 mainly regulated CD4^+^ T cell differentiation and expression of chemokine receptors, CCR4 and CXCR3, in CD4^+^ T cells. A previous study showed that exosomal RNF157 mRNA from prostate cancer cells contributes to M2 macrophage polarization 33. These studies indicated that RNF157 might play different roles in different cell types.

HDAC1 plays an important role in various biological processes, and ubiquitination is a key mechanism for regulating HDAC1 activation. In particular, TRIM46 is an ubiquitin ligase that targets HDAC1 for ubiquitination and degradation to regulate genes involved in DNA replication and repair 43. Another E3 ubiquitin ligase, CHFR, inhibits immune-resistance, drug-resistance, and stem-like phenotype in tumor cells through promoting ubiquitin-mediated degradation of HDAC1^44^. MDM2 E3 ligase mediates ubiquitination and degradation of HDAC1 in vascular calcification 45. Although HDAC1 plays a vital role in Th cell function and differentiation 14, its regulation through ubiquitination in CD4^+^ T cells remains to be clarified. In this study, we found that RNF157 is a key ubiquitination regulator of HDAC1. RNF157 promoted Th1 differentiation and attenuated Th17 differentiation and CCR4 and CXCR3 expression through regulating HDAC1 ubiquitination and degradation. Furthermore, the absence of RNF157 led to higher steady state levels of HDAC1 but similar degradation kinetics, which would appear to indicate a more complex mechanism of RNF157 action. It is imperative to further investigate whether RNF157 also regulates HDAC1 degradation through other mechanisms. Previous study showed that loss of HDAC1 in CD4^+^ T cells almost completely abrogates EAE induction 17; however, our results showed that *Hdac1^CKO^* mice were not completely protected from EAE. Nonetheless, the highly attenuated EAE observed in absence of HDAC1 provides an insufficiently robust environment to test the role of RNF157. A more definitive experiment would be testing the ability of RNF157 loss to increase EAE development in mice carrying a ubiquitinylation-resistant allele of HDAC1, such as HDAC1^K74/78R^. Such mice should be susceptible to EAE but unaffected by loss of RNF157. A previous study reported that exosomal RNF157 mRNA from prostate cancer cells contributes to M2 macrophage polarization by destabilizing HDAC1^33^, which indicates that the RNF157-HDAC1 complex plays an important role in various biological functions. In addition, several other proteins were predicted to bind RNF157. Although other proteins were less documented in the regulation of CD4^+^ T cell functions, their possible regulatory functions warrant further studies. It is imperative to further investigate whether RNF157 also regulates CD4^+^ T cell differentiation through regulating these molecules.

In summary, we identified RNF157 as a vital regulator of CD4^+^ T cell differentiation; it promoted Th1 differentiation, but attenuated Th17 differentiation and expression of CCR4 and CXCR3 in CD4^+^ T cells in a T cell-intrinsic manner through promoting the ubiquitination and degradation of HDAC1, thereby limiting EAE development. Notably, RNF157 expression was significantly decreased, and showed a significant negative correlation with RORγt expression in MS. These results suggest that RNF157 serves as a potential target for treating adaptive immune responses driving MS and other autoimmune disorders.

## Supplementary Material

Supplementary figures and tables.
