# Supplementary material for: RNF157 attenuates CD4+ T cell-mediated autoimmune response by promoting HDAC1 ubiquitination and degradation
Source: Theranostics. 2023 Jun 19;13(11):3509–23. doi: 10.7150/thno.86307 (PMC10334825; doi:10.7150/thno.86307)
Supplement: Supplementary file 1 — Supplementary figures and tables. [file thnov13p3509s1.pdf]

RNF157 attenuated $CD4^+$ T cell-mediated autoimmune response by promoting HDAC1 ubiquitination and degradation

Peng Wang[1#], Jingjing Zhao[2, 3#], Yunke Tan[1], Junli Sheng[4], Shitong He[4], Yitian Chen[4], Dingnai Nie[4], Xiaolong You[4], Jinmei Luo[5]*, Yanling Zhang[6]*, Shengfeng Hu[4, 7]*

[1]Department of Emergency Medicine, Sun Yat-sen Memorial Hospital, Sun Yat-sen University, Guangzhou, China.

[2]Department of Biotherapy, Sun Yat-sen University Cancer Center, Guangzhou, China.

[3]State Key Laboratory of Oncology in South China, Collaborative Innovation Center for Cancer Medicine, Sun Yat-sen University Cancer Center, Guangzhou, China.

[4] The Second Affiliated Hospital, The State Key Laboratory of Respiratory Disease, Guangdong Provincial Key Laboratory of Allergy & Clinical Immunology, Guangzhou Medical University, Guangzhou, China.

[5]Department of Internal Medicine, Medical Intensive Care Unit and Division of Respiratory Diseases, the Third Affiliated Hospital of Sun Yat-sen University, Guangzhou, China.

[6]Experimental Center of Teaching and Scientific Research, School of Laboratory Medicine and Biotechnology, Southern Medical University, Guangzhou, China.

[7]Department of Rheumatology and Clinical Immunology, Zhujiang Hospital, Southern Medical University, Guangzhou, China.

# These authors contributed equally to this work.

*** Corresponding author:**

Shengfeng Hu, M.D., Ph.D. E-mail: hushengfeng@gzhmu.edu.cn

Yanliang Zhang, M.D., Ph.D. E-mail: drzyl@smu.edu.cn

Jinmei Luo, M.D., Ph.D. E-mail: luojm3@mail.sysu.edu.cn

Supplementary figures and table

Figure S1

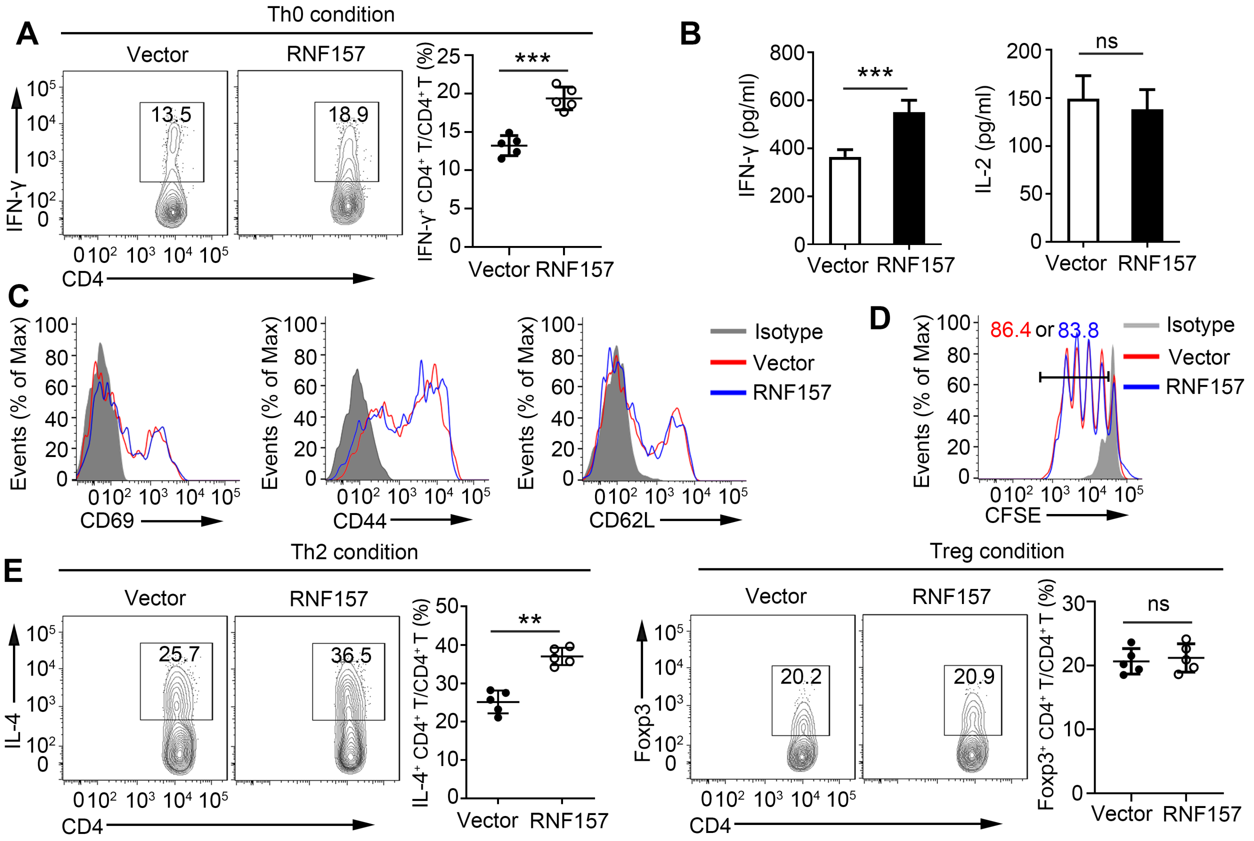

Figure S1, Related to Figure 1. RNF157 is involved in human CD4$^+$ T cell differentiation. Purified human naïve CD4$^+$ T cells were isolated, infected with control retrovirus (Vector) or retrovirus expressing RNF157, and then stimulated with plate bound anti-CD3 plus anti-CD28 (Th0), or under standard Th2 conditions or Treg conditions for 5 days. **(A)** Flow cytometry of intracellular IFN-γ. Pooled data are presented in the below panel. **(B)** Concentration of IFN-γ and IL-2 in cultural supernatant was measured by ELISA. **(C)** Expression of activation markers by CD4$^+$ T cells were determined. **(D)** CD4$^+$ T cells were labeled with CFSE, stimulated and determined by flow cytometry. **(E)** Flow cytometry of intracellular IL-4 or Foxp3 in human CD4$^+$ T cells. Pooled data are presented in the below panel. Data shown are the mean ±SD. $^{**}P < 0.01$, $^{***}P < 0.001$, ns, no significant difference by an unpaired *t*-test. Data are representative of three independent experiments with similar results.

Figure S2

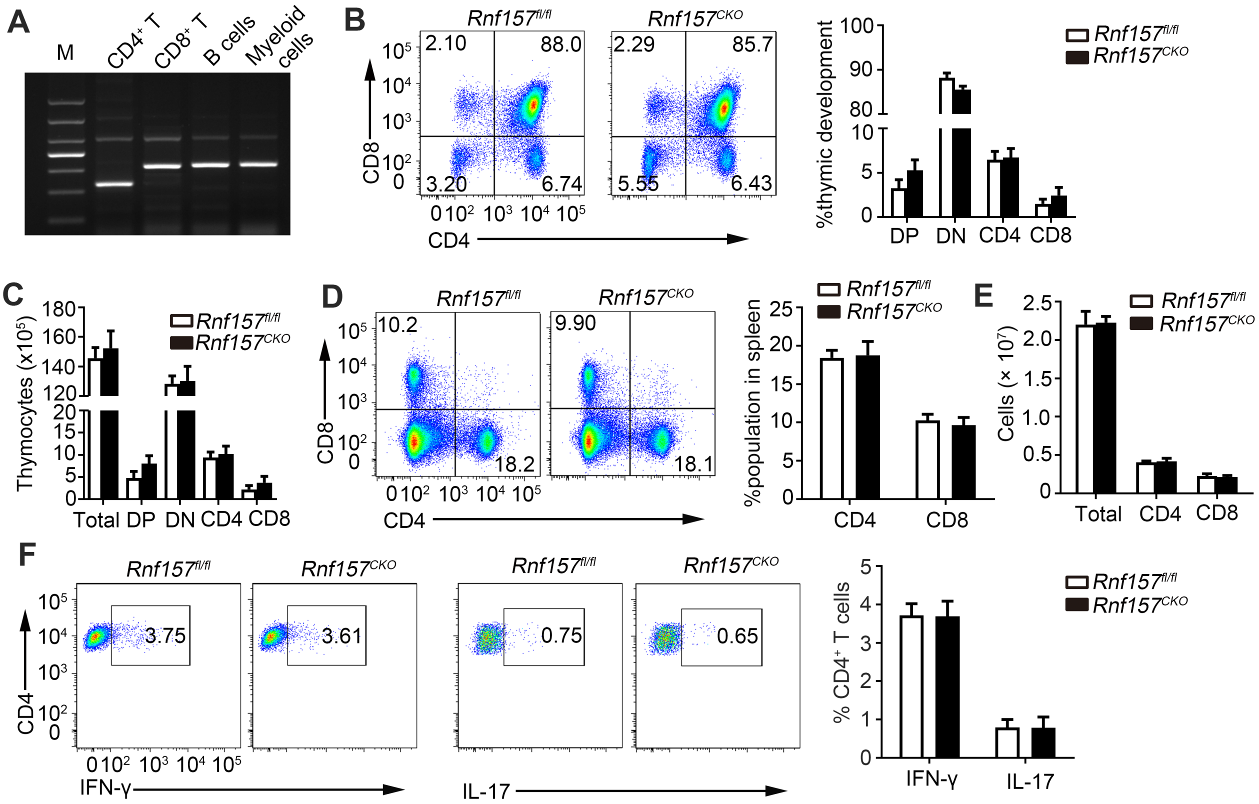

Figure S2, Related to Figure 2. RNF157 did not affect thymic development. (A) CD4$^+$ T (CD3$^+$ CD4$^+$), CD8$^+$T (CD3$^+$ CD8$^+$) and B cells (CD19$^+$) were sorted from spleens of *Rnf157*^{CKO} mice by flow cytometry, and myeloid cells were isolated from bone marrow of *Rnf157*^{CKO} mice. The knockout efficiency of RNF157 was examined by PCR. **(B)** Representative expression of CD4 and CD8 from thymocytes of *Rnf157*$^{fl/fl}$ and *Rnf157*^{CKO} mice (3 weeks old). Pooled data are presented in the right panel. DN, double negative (CD4$^-$ CD8$^-$); DP, double positive (CD4$^+$ CD8$^+$); CD4, CD4 single positive (CD4$^+$ CD8$^-$); CD8, CD8 single positive (CD4$^-$ CD8$^+$). **(C)** Total numbers of thymocytes in each stage of thymic development (n = 3 mice per genotype). **(D)** Representative expression of CD4$^+$ T and CD8$^+$ T (Gated in CD3$^+$ T cells) from splenocytes of *Rnf157*$^{fl/fl}$ and *Rnf157*^{CKO} mice (8-10 weeks old). **(E)** Total numbers (right) of CD4$^+$ T and CD8$^+$ T (Gated in CD3$^+$ T cells) from splenocytes of *Rnf157*$^{fl/fl}$ and *Rnf157*^{CKO} mice (n = 3 mice per genotype). **(F)** Splenocytes were brief stimulated with PMA/ionomycin ex vivo and the intracellular production of IFN-γ

and IL-17 by $CD4^+$ T cells was determined. Pooled data are presented in the right panel. Data shown are the mean ±SD. Data are representative of three independent experiments with similar results.

Figure S3

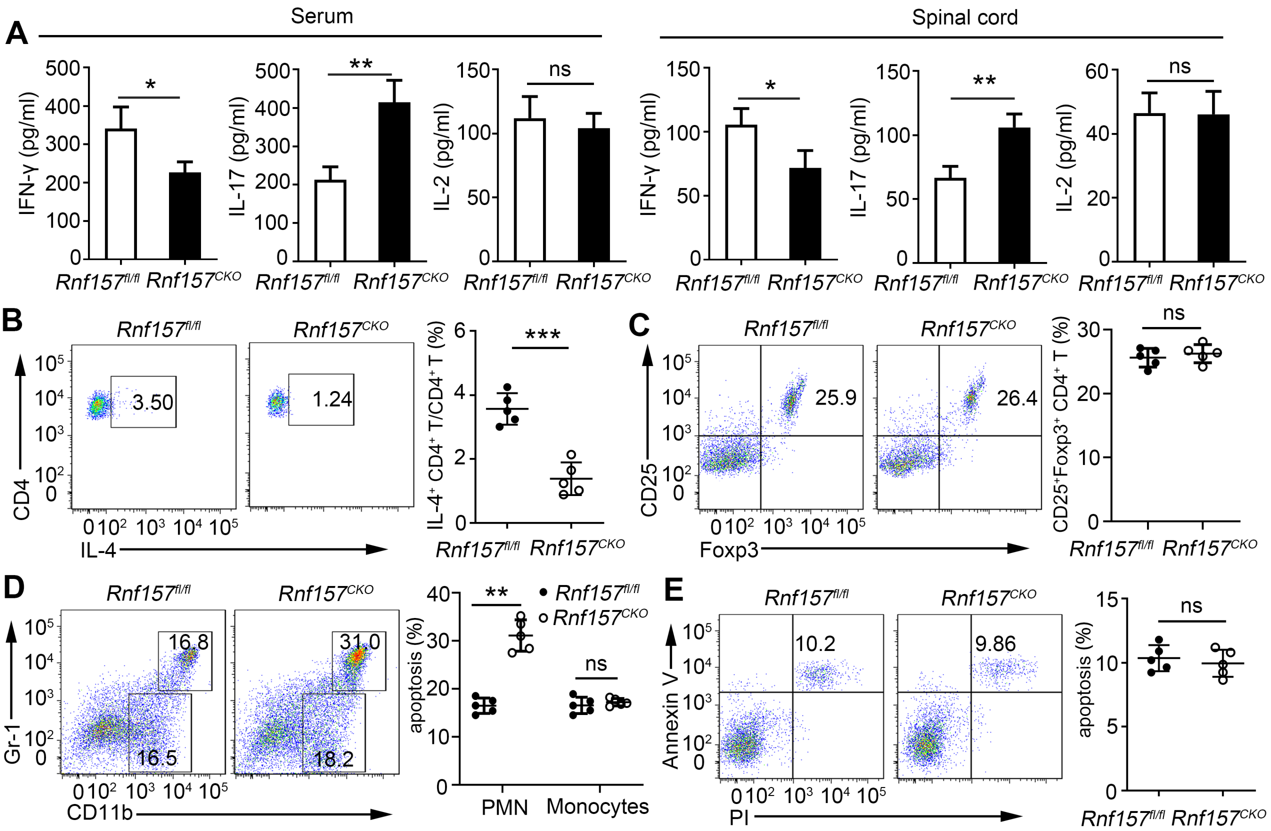

Figure S3, Related to Figure 2. RNF157 deficiency in $CD4^+$ T cells regulated $CD4^+$ T cell differentiation during EAE development. *Rnf157*$^{fl/fl}$ and *Rnf157*^{CKO} mice were immunized with MOG(35-55) peptide in CFA adjuvant and pertussis toxin (PTX) to induce EAE. **(A)** Mice were harvested on day 28, and concentration of IFN-γ, IL-17, and IL-2 in serum and spinal cord was measured by ELISA. **(B)** The cells from the central nervous system (the spinal cord and brain) were restimulated directly ex vivo and the intracellular production of IL-4 by $CD4^+$ T cells was determined. Pooled data are presented in the right panel. **(C)** Expression of CD25 and Foxp3 were detected on $CD4^+$ T cells from the central nervous system. Pooled data are presented in the right panel. **(D)** Ratios of neutrophils ($CD11b^+$ $Gr\text{-}1^+$) or monocytes ($CD11b^+$ $Gr\text{-}1^+$) in the CNS. **(E)** Identification of apoptosis among $CD4^+$ T cells from the central nervous system by flow cytometry assay of Annexin V/PI double staining. Data shown are the mean ±SD. $^*P < 0.05$, $^{**}P < 0.01$ and $^{***}P < 0.001$ by an unpaired *t*-test. Data are representative of three independent experiments with similar results.

Figure S4

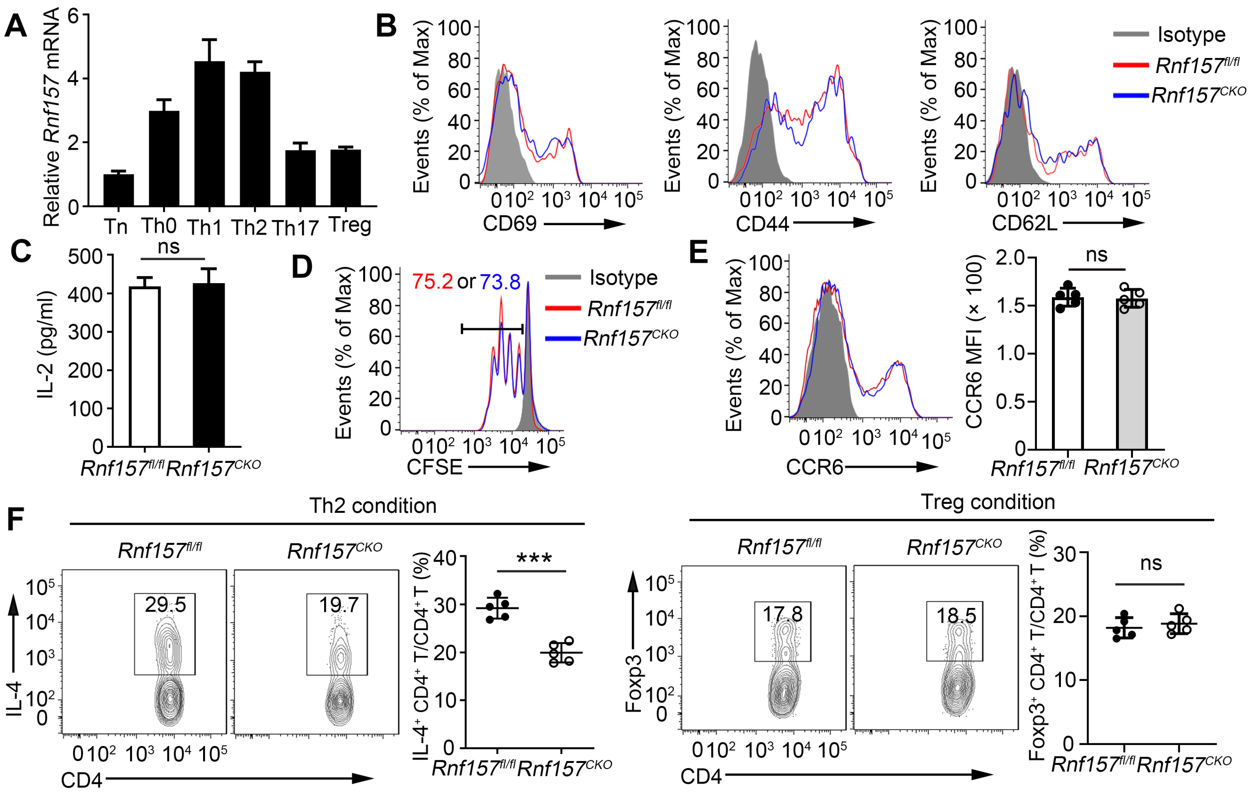

Figure S4, Related to Figure 3. RNF157 deficiency in CD4$^+$ T cells regulated CD4$^+$ T cell differentiation *in vitro*. Purified naïve CD4$^+$ T cells from *Rnf157$^{fl/fl}$* and *Rnf157^{CKO}* mice were isolated, and stimulated with anti-CD3 plus anti-CD28 (Th0) **(B-E)**, or under standard Th1, Th2, Th17 or Treg conditions, and harvested on day 5. **(A)** RNF157 mRNA expression were assessed using qPCR. **(B)** Expression of activation markers by CD4$^+$ T cells were determined. **(C)** Representative flow cytometry data showing CCR6 on CD4$^+$ T cells. Pooled data of mean fluorescence intensity (MFI) are presented in the right panel. **(D)** Concentration of IL-2 in cultural supernatant was measured by ELISA. **(E)** CD4$^+$ T cells were labeled with CFSE, stimulated and determined by flow cytometry. **(F)** Flow cytometry of intracellular IL-4 or Foxp3 in CD4$^+$ T cells. Pooled data are presented in the below panel. Data shown are the mean ±SD. *P < 0.05, **P < 0.01 and ***P < 0.001 by an unpaired t-test. Data are representative of three independent experiments with similar results.

Figure S5

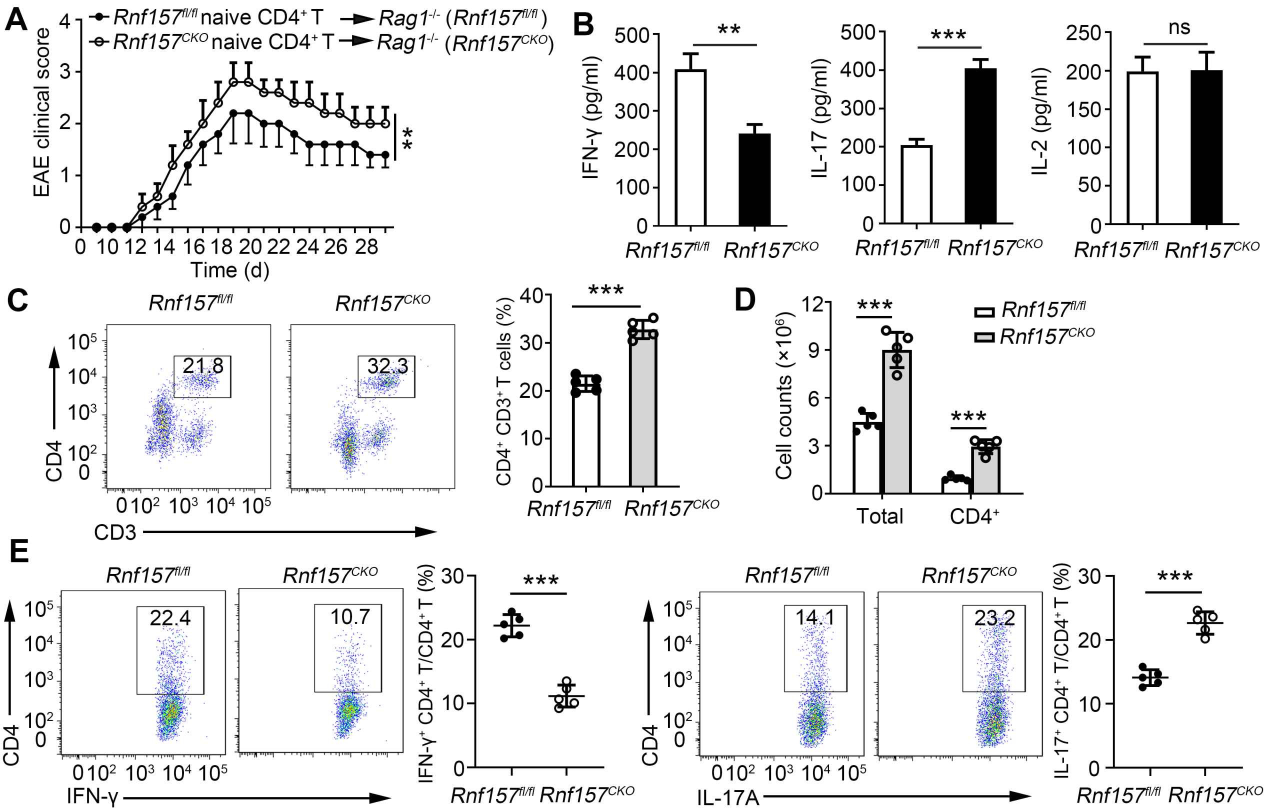

Figure S5, Related to Figure 4. Purified *Usp1*$^{fl/fl}$ or *Usp1*^{CKO} naïve CD4$^+$ T cells were adoptively transferred into *Rag1*$^{-/-}$ mice. Recipient mice were immunized with MOG(35-55) peptide in CFA adjuvant and pertussis toxin to induce EAE. **(A)** The graph shows the clinical score of EAE (n = 5 respectively). **(B)** Mice were harvested on day 28, and concentration of IFN-γ, IL-17, and IL-2 in serum was measured by ELISA. **(C)** Percentage of CD4$^+$ T cells among cells infiltrating to the central nervous system was analyzed by flow cytometry and pooled data are presented in the right panel. **(D)** Total number of cells and CD4$^+$ T cells infiltrating the central nervous system. **(E)** The cells from the central nervous system (the spinal cord and brain) were restimulated directly ex vivo and the intracellular production of IFN-γ and IL-17A by CD4$^+$ T cells was determined. Pooled data are presented in the right panel. Data shown are the mean ±SD. *$P < 0.05$, **$P < 0.01$ and ***$P < 0.001$ by an unpaired *t*-test. Data are representative of three independent experiments with similar results.

Figure S6

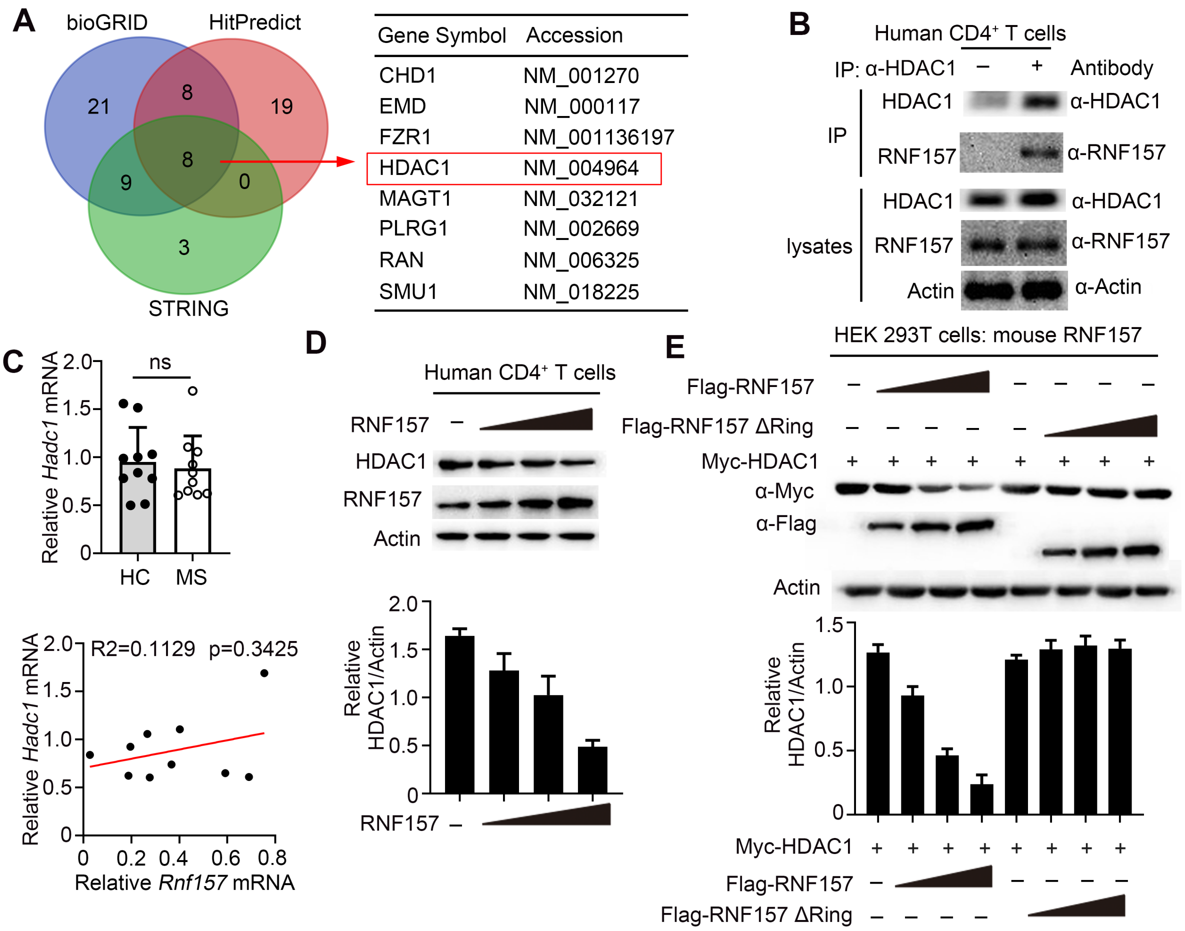

Figure S6, Related to Figure 5. RNF157 promoted the degradation of HDAC1. (A) Proteins with binding potential to human RNF157 were predicted on BioGRID, HitPredict and STRING. **(B)** Immunoprecipitation (IP) and immunoblot (IB) analysis of human CD4+T cells stimulated with anti-CD3 plus anti-CD28 for 3 hours. **(C)** HDAC1 mRNA expression were assessed using qPCR analysis in CD4^{+} T cells from MS and HC. And the correlation of the expression of RNF157 with that of T-bet, RORγt, and Foxp3 in CD4^{+} T cells from HC (n = 10) and MS (n =10); results were plotted and analyzed with the linear-regression *t*-test. **(D)** Purified human naïve CD4^{+} T cells were isolated, infected with control retrovirus (-) or increasing doses of retrovirus exrepssiong Flag-RNF157 (wedge), and then stimulated with anti-CD3 and anti-CD28 for 3 hours. HDAC1 IB analysis were then carried out. **(E)** IB analysis of HEK293T cells transfected with Myc-HDAC1 and increasing doses of expression vector for Flag-RNF157 (wedge) or Flag-RNF157 deleted RING domain (aa271-330).

Densitometry quantification of band intensity is presented in the below panel. Data shown are the mean ±SD. Data are representative of three independent experiments with similar results.

Figure S7

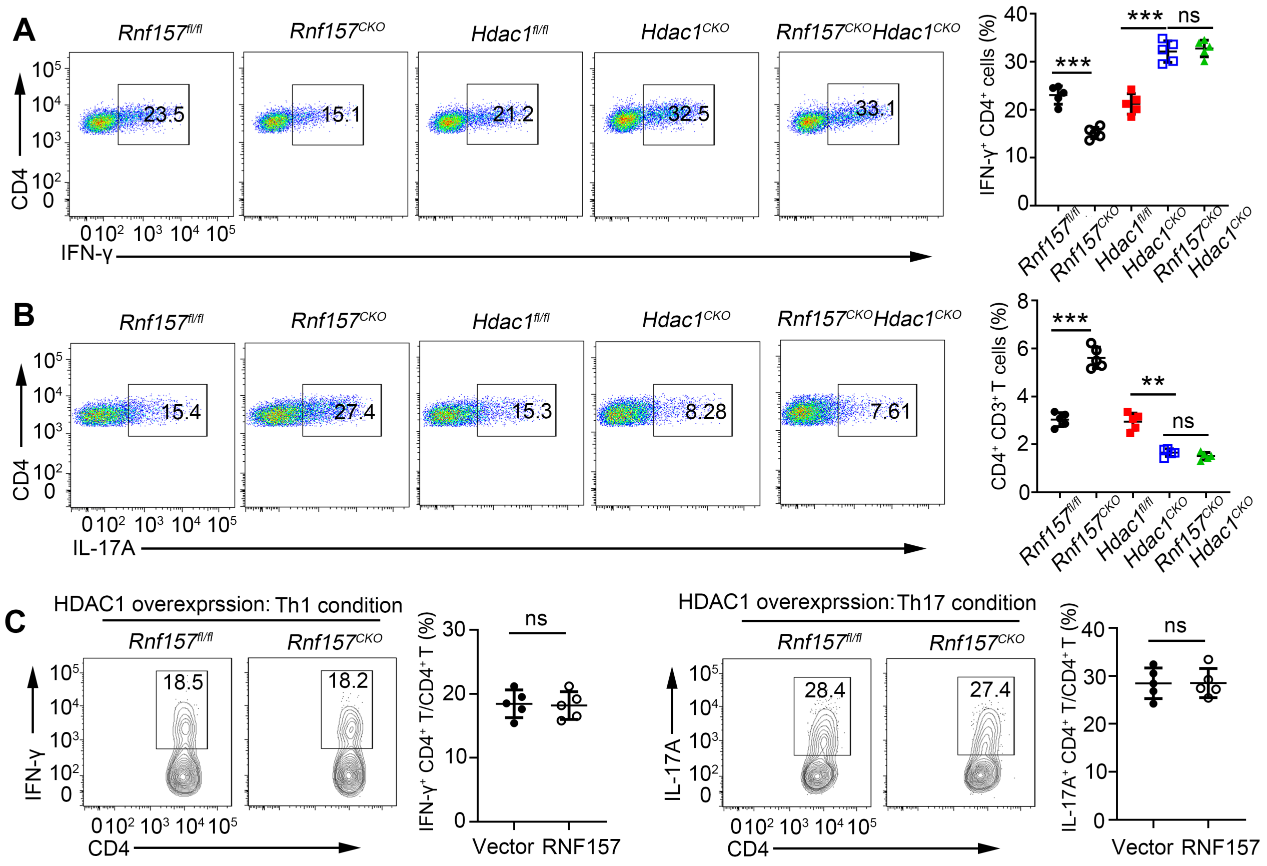

Figure S7, Related to Figure 7. *Rnf157*$^{fl/fl}$, *Rnf157*^{CKO}, *Hdac1*$^{fl/fl}$, *Hdac1*^{CKO} and *Rnf157*^{CKO} *Hdac1*^{CKO} mice were immunized with MOG(35-55) peptide in CFA adjuvant and pertussis toxin (PTX) to induce EAE. The cells from the central nervous system (the spinal cord and brain) were restimulated directly ex vivo and the intracellular production of IFN-γ **(A)** and IL-17A **(B)** by CD4^{+} T cells was determined. Pooled data are presented in the right panel. **(C)** Flow cytometry and of intracellular IFN-γ or IL-17A in *Rnf157*^{CKO} naive CD4^{+} T cells infected with retrovirus expressing HDAC1 and differentiated under standard Th1 conditions or Th17 conditions. Data shown are the mean ±SD. **$P < 0.01$ and ***$P < 0.001$ by an unpaired *t*-test. Data are representative of three independent experiments with similar results.

Table S1. Antibodies

Antigen	Reactivity	Label	Clone	Manufacture	Use
CD3	M	APC-eFluor 780	145-2C11	eBioscience	FCM
CD4	M	Percp-Cy5.5	RM4-5	eBioscience	FCM
CD8	M	APC	53-6.7	eBioscience	FCM
IFN-γ	M	eFluor 450	XMG1.2	eBioscience	FCM
IL-17A	M	FITC	eBio17B7	eBioscience	FCM
Foxp3	M	FITC	FJK-16s	eBioscience	FCM
CD25	M	PE	PC61.5	eBioscience	FCM
CD69	M	PE	H1.2F3	eBioscience	FCM
CD44	M	FITC	IM7	eBioscience	FCM
CD62L	M	PE	MEL-14	eBioscience	FCM
CD45.1	M	PE-Cy7	A20	eBioscience	FCM
CD45.2	M	APC	104	eBioscience	FCM
CD3	H	APC	OKT3	eBioscience	FCM
CD4	H	FITC	RPA-T4	eBioscience	FCM
IFN-γ	H	PE	4S.B3	eBioscience	FCM
IL-17A	H	PE-Cy7	eBio64DEC17	eBioscience	FCM
Foxp3	H	Percp-Cy5.5	PCH101	eBioscience	FCM
CD69	H	PE	FN50	eBioscience	FCM
CD44	H	FITC	SFF-2	eBioscience	FCM
CD62L	H	eFluor 450	DREG56	eBioscience	FCM
HDAC1	H/M		D5C6U	CST	WB
HDAC1	H/M		10E2	CST	WB
Ubiquitin	H/M		P4D1	CST	WB
K48-linkage Specific Polyubiquitin	All		D9D5	CST	WB
K63-linkage Specific Polyubiquitin	All		D7A11	CST	WB

FLAG	H/M		D6W5B	CST	WB
HA	H/M		C29F4	CST	WB
Myc	H/M		9B11	CST	WB
β-Actin	H/M		D6A8	CST	WB

M, Mouse; H, Human

Table S2. Gene-specific primers used for qRT-PCR

Gene	Forward primer	Reverse primer
hRnf7	AGGCGACAAGATGTTCTCCCTC	TCAGCTTGACATCTAAGACAGGC
hRnf19a	GGAGTCTGTCAGGAAGTGCCAT	CCAAGCTGACTGTGCCAGATTC
hRnf157	CTCACCTTGTCGTCATCTGGAG	AGACGGTGTCAGTGCTGATCTG
hRnf169	CAGACACATCGCTCGGCATTTG	GGCTTTGTTGCCTGGAACTGCT
hRnf213	GGAAAGGAAACCTCTGAACTCGG	CTCGTTCTGGTCTCTGAGCATG
hRnf214	CAGTTCGTTCCAAACAGGAGTGG	CTTGGCTCCGTTCCTCACTAAC
hTbx21	ATTGCCGTGACTGCCTACCAGA	GGAATTGACAGTTGGGTCCAGG
hRorc	GAGGAAGTGACTGGCTACCAGA	GCACAATCTGGTCATTCTGGCAG
hFoxp3	GGCACAATGTCTCCTCCAGAGA	CAGATGAAGCCTTGGTCAGTGC
hHdac1	GGTCCAAATGCAGGCGATTCCT	TCGGAGAACTCTTCCTCACAGG
hGapdh	GTCTCCTCTGACTTCAACAGCG	ACCACCCTGTTGCTGTAGCCAA
mRnf157	ATCCCATGTTGCCCCTTCTG	AGCACTTGTGAAGGGAGACG
mHdac1	TGAAGCCTCACCGAATCCGCAT	TGGTCATCTCCTCAGCATTGGC
mActb	CATTGCTGACAGGATGCAGAAGG	TGCTGGAAGGTGGACAGTGAGG

M, Mouse; H, Human
